# Antiviral activity of singlet oxygen-photogenerating perylene compounds against SARS-CoV-2: Interaction with the viral envelope and photodynamic virion inactivation

**DOI:** 10.1016/j.virusres.2023.199158

**Published:** 2023-06-29

**Authors:** Petra Straková, Petr Bednář, Jan Kotouček, Jiří Holoubek, Andrea Fořtová, Pavel Svoboda, Michal Štefánik, Ivana Huvarová, Pavlína Šimečková, Josef Mašek, Daniil A. Gvozdev, Igor E. Mikhnovets, Alexey A. Chistov, Timofei D. Nikitin, Maxim S. Krasilnikov, Alexey V. Ustinov, Vera A. Alferova, Vladimir A. Korshun, Daniel Růžek, Luděk Eyer

**Affiliations:** a Laboratory of Emerging Viral Diseases, Veterinary Research Institute, CZ-621 00 Brno, Czech Republic; b Institute of Parasitology, Biology Centre of the Czech Academy of Sciences, CZ-370 05 České Budějovice, Czech Republic; c Department of Experimental Biology, Faculty of Science, Masaryk University, CZ-62500 Brno, Czech Republic; d Faculty of Science, University of South Bohemia, Ceske Budejovice, CZ-37005, Czech Republic; e Department of Pharmacology and Toxicology, Veterinary Research Institute, CZ-621 00 Brno, Czech Republic; f Department of Pharmacology and Pharmacy, Faculty of Veterinary Medicine, University of Veterinary Sciences Brno, CZ-612 42 Brno, Czech Republic; g Department of Chemistry and Biochemistry, Mendel University in Brno, CZ-61300 Brno, Czech Republic; h Department of Biology, Lomonosov Moscow State University, Moscow, 119991, Russia; i Department of Chemistry, Lomonosov Moscow State University, Moscow, 119991, Russia; j Shemyakin-Ovchinnikov Institute of Bioorganic Chemistry, Moscow, 117997, Russia

**Keywords:** SARS-CoV-2, Perylene-related compound, Antiviral activity, Membrane, Liposome, Photodynamic inactivation

## Abstract

The coronavirus disease 2019 (COVID-19) pandemic, caused by severe acute respiratory syndrome coronavirus 2 (SARS-CoV-2), has prompted great interest in novel broad-spectrum antivirals, including perylene-related compounds. In the present study, we performed a structure–activity relationship analysis of a series of perylene derivatives, which comprised a large planar perylene residue, and structurally divergent polar groups connected to the perylene core by a rigid ethynyl or thiophene linker. Most of the tested compounds did not exhibit significant cytotoxicity towards multiple cell types susceptible to SARS-CoV-2 infection, and did not change the expressions of cellular stress-related genes under normal light conditions. These compounds showed nanomolar or sub-micromolar dose-dependent anti-SARS-CoV-2 activity, and also suppressed the *in vitro* replication of feline coronavirus (FCoV), also termed feline infectious peritonitis virus (FIPV). Perylene compounds exhibited high affinity for liposomal and cellular membranes, and efficiently intercalated into the envelopes of SARS-CoV-2 virions, thereby blocking the viral–cell fusion machinery. Furthermore, the studied compounds were demonstrated to be potent photosensitizers, generating reactive oxygen species (ROS), and their anti-SARS-CoV-2 activities were considerably enhanced after irradiation with blue light. Our results indicated that photosensitization is the major mechanism underlying the anti-SARS-CoV-2 activity of perylene derivatives, with these compounds completely losing their antiviral potency under red light. Overall, perylene-based compounds are broad-spectrum antivirals against multiple enveloped viruses, with antiviral action based on light-induced photochemical damage (ROS-mediated, likely singlet oxygen-mediated), causing impairment of viral membrane rheology.

## Highlights

• Perylene compounds show antiviral activity against SARS-CoV-2 and feline coronavirus.
• Replication of multiple SARS-CoV-2 subvariants is suppressed by perylene compounds.
• Perylene compounds block the fusion of SARS-CoV-2 with the host cell.
• Expressions of cellular stress-related genes is not affected by perylene compounds.
• Photosensitization is the major mechanism underlying their anti-SARS-CoV-2 activity.

## Graphical abstract

**Figure:**
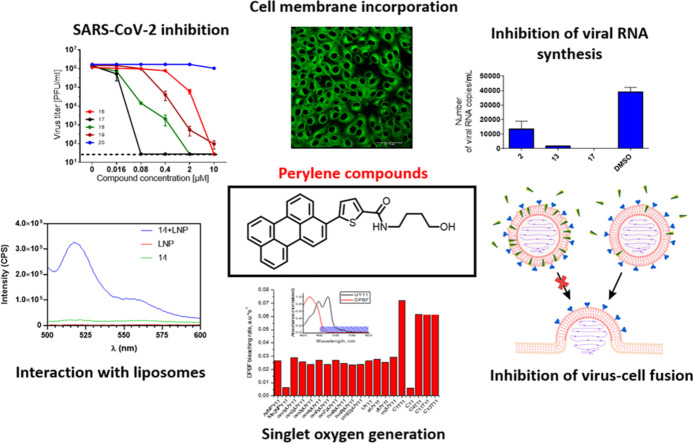

## 1 Introduction

Severe acute respiratory syndrome coronavirus 2 (SARS-CoV-2), a member of the *Coronaviridae* family, is an enveloped virus with a non-segmented +ssRNA genome of approximately 30 kb. SARS-CoV-2 was first discovered in 2019 in China, and later confirmed to be the causative agent of coronavirus disease 2019 (COVID-19) (Tu et al., 2020). The virus spread rapidly and widely, causing a global pandemic with nearly 750 million confirmed cases and over 6 million reported deaths to date (https://covid19.who.int/, accessed on 30 January 2023). Most cases of SARS-CoV-2 infection are asymptomatic or relatively mild. However, in some cases, particularly among elderly patients and those with cardiac and respiratory disease, the infection can lead to severe and life-threatening acute respiratory distress syndrome (ARDS) or pneumonia, potentially resulting in sepsis, multiple organ dysfunction, and death (Paraskevis et al., 2020). The urgent need to reduce COVID-19 severity and related mortality prompted the massive repurposing of approved antivirals in efforts to use them against SARS-CoV-2. However, this approach has had limited success (Martinez, 2022; Punekar et al., 2022). The major medical impact of the SARS-CoV-2 pandemic made it an obvious research priority to develop new and effective broad-spectrum antivirals that suppress critical phases of the viral replication cycle. In general, the development of broad-spectrum antivirals would be appropriate preparation to combat potential future epidemics/pandemics from new emerging viruses (Bekerman and Einav, 2015; Ianevski et al., 2022; Mercorelli et al., 2018).

Two decades ago, researchers discovered the antiviral activity of derivatives of perylene, a pentacyclic aromatic hydrocarbon (Alferova et al., 2022). Subsequent reports described their broad-spectrum activity against medically important viruses, and abilities to target viral lipid envelopes and cell membranes, and these compounds were termed rigid amphipathic fusion inhibitors (RAFIs) (St.Vincent et al., 2010). Structurally, RAFIs are amphipathic compounds with inverted-cone molecular geometry. They comprise hydrophilic “heads”, along with planar hydrophobic “tails” that have a smaller diameter and an arylethynyl core (Colpitts et al., 2013). From a mechanistic perspective, it has been proposed that RAFIs have two modes of action. In one, it is postulated that RAFIs become incorporated into the hydrophobic environments of viral envelopes, thereby disrupting viral membrane rheology and impairing the positive-to-negative membrane curvature transition, which is crucial for the virus–cell fusion process. This type of antiviral effect is based on biophysical (not biochemical) mechanisms and is highly dependent on the molecule's rigidity, amphipathicity, and geometry (Speerstra et al., 2018; St.Vincent et al., 2010). Another reported mechanism of action for RAFIs is based on their action as photosensitizers and the light-dependent oxidation of viral lipids through the generation of singlet oxygen (^1^O_2_) targeting the C=C double bonds of unsaturated phospholipids in viral membranes . The accumulation of oxidized lipids in viral envelopes promotes increased membrane rigidity and prevents the membrane curvature transition during virus–cell fusion (Vigant et al., 2014). Since membranes are a highly evolutionarily conserved structural element for all enveloped viruses, and because membranes are not linked to the viral genomes through the replication-transcription-translation machinery, RAFIs have the potential to be broad-spectrum antiviral agents with an extremely high resistance barrier (St.Vincent et al., 2010). Targeting the viral lipid membrane is an important recent trend in the development of antivirals and vaccines (Correa Sierra and Schang, 2022; Park and Cho, 2023; Sardar et al., 2022). Although both suggested mechanisms involve the inhibition of virion-cell fusion, the name RAFI emphasizes the molecule's geometric shape and polarity, not the presence of a photosensitizing chromophore. Therefore, we will henceforth refer to these compounds as perylene derivatives.

In the present study, we investigated the *in vitro* anti-SARS-CoV-2 activity and cytotoxicity of a series of known antiviral perylene derivatives, in Vero cells and human Caucasian colon adenocarcinoma (CaCo-2) cells. To facilitate analysis of the structure–activity relationship (SAR), we focused on compounds possessing bulky hydrophobic perylenylethynyl or perylenylthienyl groups and chemically distinct polar moieties. The compounds that exhibited the highest viral inhibition potency and low cytotoxicity were further investigated to elucidate the mechanisms of their anti-SARS-CoV-2 action. To demonstrate the compounds’ interactions with viral, cellular, and liposomal membranes, respectively we used specific antiviral cell-based assays, confocal microscopy, and spectrofluorometric measurements. Additionally, we studied the photodynamic inactivation of SARS-CoV-2 virions to demonstrate that the antiviral activity of amphipathic perylene compounds depended on light and singlet oxygen generation. Finally, we profiled the compounds’ cytotoxic effects on nuclear receptor signaling, endogenous metabolism disruption, and induction of cell stress, to exclude potential deleterious effects of the perylene derivatives on cell proliferation and viability. Our results suggested that photodynamic lipid oxidation was the main and decisive mechanism underlying the anti-SARS-CoV-2 activity of amphipathic perylene photosensitizers, and that the compounds lost their antiviral activity under red light, even at high (two-digit micro-molar) concentrations.

## 2 Material and methods

### 2.1 Compounds, viruses, and cells

**Fig. 1:**
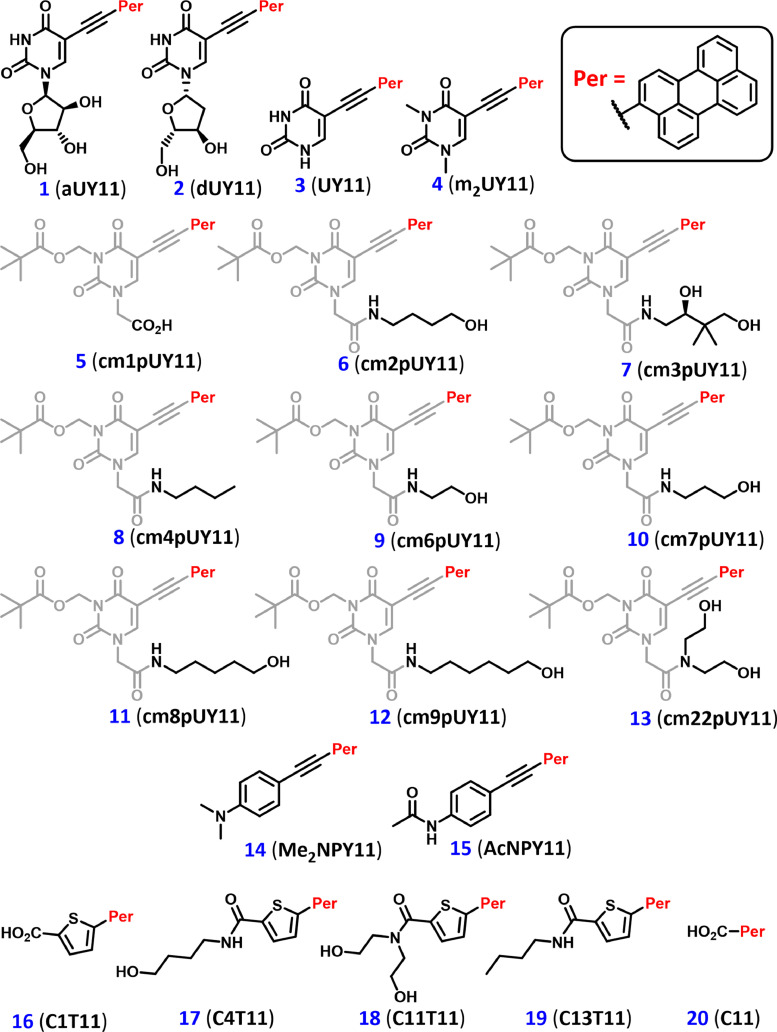
Structures of the compounds used in this study. (For interpretation of the references to color in this figure legend, the reader is referred to the web version of this article.)

Twenty structurally divergent perylene derivatives (Fig. 1) were used to evaluate anti-SARS-CoV-2 activity and cytotoxicity, and to elucidate the molecular mechanism of action. Compounds **1** (Chistov et al., 2017; St.Vincent et al., 2010), **2** (Andronova et al., 2003), **3, 14**–**19** (Slesarchuk et al., 2020), **4** (Chistov et al., 2023), **5**–**13** (Chistov et al., 2019), and **20** (Astakhova et al., 2008) were prepared as previously described. All compounds were dissolved in 100% DMSO to prepare a 10 mM stock solution, and stored in the dark at −20 °C. Before use, the compounds were diluted in growth medium to obtain the concentrations required for the antiviral, cytotoxicity, and mechanistic assays.

The following variants of SARS-CoV-2 were used for our antiviral studies: SARS-CoV2/human/Czech Republic/951/2020 (variant Wuhan), hCoV-19/Czech Republic/NRL_240/2021 EPI_ISL_850,685 (variant B.1.1.7, Alpha), hCoV-19/Czech Republic/NRL_7112/2021 EPI_ISL_2,357,740 (variant B.1.351, Beta), hCoV-19/Czech Republic/NRL_8413/2021 EPI_ISL_2,562,083 (variant P10.16, Gamma), hCoV-19/Czech Republic/NRL_7102/2021 EPI_ISL_2,357,738 (variant B1.617.2, Delta), and p2, isolate 17,577/21 (variant B.1.1.529, Omicron). All variants had been isolated from clinical samples at the National Institute of Health, Prague, Czech Republic), and were kindly provided by Dr. Jan Weber, Institute of Organic Chemistry and Biochemistry, Prague, Czech Republic. Prior to use, the viruses were passaged five times through Vero E6 cells (ATCC CRL-1586) for multiplication. In our antiviral studies, we also used feline coronavirus (FCoV), also referred to as feline infectious peritonitis virus (FIPV, ATCC VR990), for comparison as an important veterinary pathogen. Experiments using authentic SARS-CoV-2 and FIPV were performed in our BSL3 and BSL2 facilities, respectively.

Vero cells (ATCC CCL-81, African Green Monkey, adult kidney, epithelial), Vero E6 cells (ATCC CRL-1586), human Caucasian colon adenocarcinoma cells (CaCo-2, ATCC HTB-37), and *Felis catus* kidney cortex cells (CRFK, ATCC CCL-94) were grown in Dulbecco's modified Eagle's medium (DMEM) supplemented with 10% (Vero, Vero E6, and CRFK) or 20% (CaCo-2) newborn calf serum plus 100 U/mL penicillin, 100 µg/mL streptomycin, and 1% glutamine (Sigma-Aldrich, Prague, Czech Republic), and cultured at 37 °C under 5% CO_2_. Vero (ATCC CCL-81), CaCo-2 (ATCC HTB-37), and CRFK (ATCC CCL-94) cells were used for antiviral and cytotoxicity assays, and Vero E6 cells (ATCC CRL-1586) were used for plaque assays.

### 2.2 Cytotoxicity assays

To determine the overall cytotoxicity of the tested compounds, Vero cells were cultured for 24 h in 96-well plates to form a confluent monolayer, and then were treated with the tested compounds at concentrations of 2 and 10 µM. After 48 h of cultivation in the dark at 37 °C under 5% CO_2_, the cell culture medium was aspirated. The potential cytotoxicity of the tested perylene derivatives was determined based on cell viability using Cell Counting Kit-8 (Dojindo Molecular Technologies, Munich, Germany) according to the manufacturer's instructions. The cell viabilities of compound-treated Vero cells were compared with those of control DMSO-treated cells. We also used the above-described protocol to assess the cytotoxicity of the selected perylene antivirals, at concentrations ranging from 0 to 10 µM, in CaCo-2 and CRFK cells.

### 2.3 Viral titer inhibition assay

**Fig. 2:**
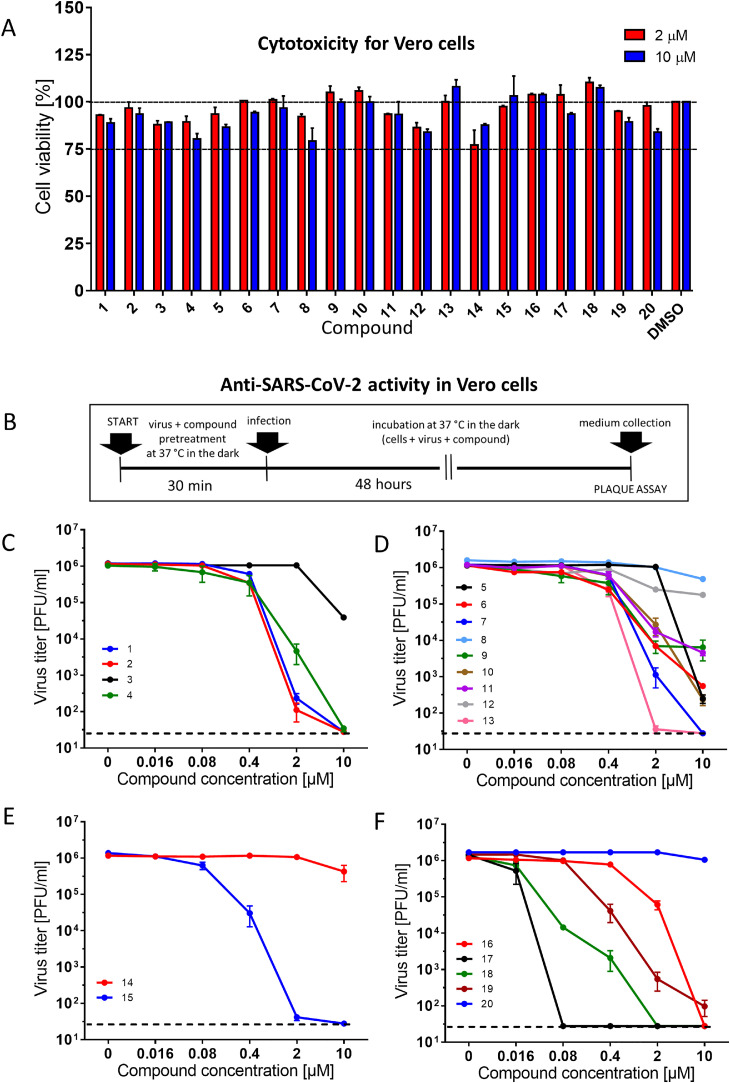
Cytotoxicity and anti-SARS-CoV-2 activity of the tested compounds. (A) The compounds’ cytotoxicity towards Vero cells was determined at concentrations of 2 and 10 μM, and expressed as percentage of cell viability. Vero cells were seeded in 96-well plates for 24 h, then treated with the compounds and incubated for 48 h. Horizontal dashed lines represent the 75% and 100% thresholds of cell viability. (B) Schematic representation of the antiviral assays as performed in C–F. (C–F) Inhibition of SARS-CoV-2 (Wuhan variant) replication by the indicated compounds. The virus was pretreated with the compounds (concentrations of 0–10 μM) for 30 min in the dark, and then used to infect Vero cell monolayers (MOI of 0.1). Infected cells were then incubated with the compounds for 48 h. After incubation, media supernatants were collected, and viral titers were determined by plaque assay and expressed as PFU/mL. Data are expressed as the mean ± SD of two independent experiments, each performed in triplicate. The horizontal dashed line indicates the minimum detectable threshold of 1.44 log_10_ PFU/mL. (For interpretation of the references to color in this figure legend, the reader is referred to the web version of this article.)

To measure the antiviral efficacy of perylene compounds in cell cultures, we performed a viral titer inhibition assay using Vero or CaCo-2 cells (for anti-SARS-CoV-2 assay) and CRFK cells (for anti-FIPV assay). Cells were seeded in 96-well plates (approximately 2 × 10^4^ cells per well) and incubated for 24 h to form a confluent monolayer. In a separate microtiter plate, the virus in DMEM (MOI of 0.1) was mixed with each compound (in triplicate) and incubated in the dark at 37 °C for 30 min. Following this virus-compound pre-treatment step, we performed dose-response antiviral studies with SARS-CoV-2 (Wuhan variant) and FIPV using compound concentrations of 0, 0.016, 0.08, 0.4, 2, and 10 µM (Fig. 2B). Selected compounds were further tested at concentrations of 0, 1, and 10 µM, to evaluate their antiviral activity against SARS-CoV-2 variants Alpha (B.1.1.7), Beta (B.1.351), Gamma (p.1), Delta (B.1.617.2), and Omicron (B.1.1.529). As a negative control, DMSO was added (final concentration 0.5% v/v) to the virus- and mock-infected cells. At 48 h post-infection (p.i.), the culture medium was collected and viral titers were determined by plaque assays (expressed as PFU/mL) as previously described (Stefanik et al., 2021). Titers were normalized and converted to percent inhibition, and used to estimate the 50% effective concentration (EC_50_) (Fig. S1 and Table 1). To better describe the compounds’ antiviral (biological) potency, we also calculated EC_50_ values from log-transformed virus titers (App. EC_50_) (Fig. 2C–F and Table 1).

**Table 1:** Antiviral properties of the studied compounds.

Compound	EC_50_ [µM]^a^^,^^b^	95% confidence interval	App. EC_50_ [µM]^a^^,^^c^	95% confidence interval
***Nucleoside-derived perylene compounds***				
1 (aUY11)	0.4058	0.3677 to 0.4478	1.117	1.017 to 1.226
2 (dUY11)	0.2564	0.2140 to 0.3070	0.8430	0.7452 to 0.9535
***Aglycosilated (ribose-free) perylene compounds***				
3 (UY11)	>2.5	–	>5	–
4 (m_2_UY11)	0.4061	0.3238 to 0.5094	2.056	1.901 to 2.223
***Perylene compounds with pentose sugar replacement***				
5 (cm1pUY11)	2.048	1.713 to 2.448	>5	–
6 (cm2pUY11)	0.09147	0.05133 to 0.1630	0.7812	0.4200 to 1.453
7 (cm3pUY11)	0.4169	0.2058 to 0.8447	1.167	0.8909 to 1.529
8 (cm4pUY11)	2.048	1.713 to 2.448	>5	–
9 (cm6pUY11)	0.1395	0.08197 to 0.2375	0.7812	0.4200 to 1.453
10 (cm7pUY11)	0.3817	0.3361 to 0.4335	1.931	1.650 to 2.261
11 (cm8pUY11)	0.4329	0.3047 to 0.6150	1.072	0.9025 to 1.274
12 (cm9pUY11)	0.8125	0.4219 to 1.565	>5	–
13 (cm22pUY11)	0.2735	0.1923 to 0.3888	0.6218	0.5096 to 0.7587
***Perylene compounds with sugar-uracil replacement***				
14 (Me_2_NPY11)	>2.5	–	>5	–
15 (AcNPY11)	0.06309	0.05548 to 0.07174	0.3252	0.2135 to 0.4951
***Perylene compounds with sugar-uracil-ethynyl replacement***				
16 (C1T11)	0.5367	0.3535 to 0.8150	2.564	1.960 to 3.353
17 (C4T11)	<0.004	–	<0.004	–
18 (C11T11)	0.01905	0.01849 to 0.01961	0.1322	0.07972 to 0.2192
19 (C13T11)	0.09818	0.09059 to 0.1064	0.4698	0.3487 to 0.6330
20 (C11)	>2.5	–	>5	–

a Determined from three independent experiments.

b Expressed as a 50% reduction in viral titer and calculated from the inflexion points of sigmoidal dose-response curves using GraphPad Prism 7.04 (GraphPad Software, Inc., USA) (Fig. S1).

c Expressed as a 50% reduction in viral titer and calculated from the inflexion points of sigmoidal dose-response curves, which were obtained by a nonlinear fit of log-transformed inhibitor concentrations versus normalized log-transformed response using GraphPad Prism 7.04 (GraphPad Software, Inc., USA).

### 2.4 Immunostaining assay

We performed a cell-based coronavirus immunostaining assay using a rabbit (2019-nCoV) spike S1 antibody (1:50; Sino Biological, Duesseldorfer, Germany) and goat anti-rabbit secondary antibody conjugated with Alexa Fluor 647 (A-21245; 1:1000; Invitrogen, Carlsbad, CA, USA), as previously described (Stefanik et al., 2021).

### 2.5 Elucidation of virucidal activity of perylene compounds

To determine the virucidal activity of the perylene compounds, SARS-CoV-2 (Wuhan variant) in DMEM (titers of 10^7^, 10^6^, and 10^4^ PFU/mL) was mixed with selected compounds (10 µM), in a microtiter plate in triplicate, and incubated in the dark at 37 °C for 120 min. Then the viability of the compound-treated virus was estimated using a plaque assay, as previously described (Stefanik et al., 2021). Viral titers were expressed as PFU/mL (Fig. 5A).

### 2.6 Viral membrane interaction studies

To demonstrate that amphipathic perylene compounds interacted with the virus envelope, Vero E6 cells were seeded in 6-well plates (approximately 10^5^ cells/well) and incubated for 24 h to form a confluent monolayer. Virus inoculum (10^6^ PFU/mL, SARS-CoV-2, Wuhan variant) was pre-treated with compounds (10 µM) for 1 h at 37 °C (Fig. 5C, Assay A) or 4 °C (Fig. 5C, Assay B). Next, the pre-treated inoculum was diluted to 100 PFU/mL and used to infect Vero E6 cells in 6-well plates for 1 h at 4 °C. Following inoculation, the cell monolayers were washed three times with PBS to remove the non-adsorbed virus, and then fresh medium with 1.5% carboxymethylcellulose was added to the cells. After 5 days of incubation at 37 °C, the cell monolayers were stained with naphthalene black, and the plaque count was determined.

### 2.7 Fusion assay

In order to demonstrate that perylene compounds inhibit the virus-cell fusion process, Vero cells were seeded in 6-well plates (1.1 × 10^6^ cells/well) and incubated for 24 h to form a confluent monolayer. Then, SARS-CoV-2 (Wuhan variant, 100 PFU/mL) was added to the cells and incubated for 2 h at 4 °C. Following incubation, the cell monolayers were washed with ice-cold PBS three times to remove the non-adsorbed virus and fresh ice-cold medium with the tested compounds (0, 1, 10 µM) was added to the cells and incubated for 2 h at 4 °C. After additional 2-h incubation at 37 °C, the medium was aspirated, the cells were washed with PBS and then fresh medium with 1.5% carboxymethyl cellulose was added to the cells. After 5 days of incubation at 37 °C, the cell monolayers were stained with naphthalene black and the plaque count was determined (Fig. 5F).

### 2.8 Quantification of viral RNA synthesis

Vero cell monolayers were infected with SARS-CoV-2 (Wuhan variant, MOI of 0.1), which had previously been pre-treated with the tested compounds (at 10 µM), and incubated in the dark at 37 °C for 48 h. Next, the media supernatants or cell pellets, respectively, were analyzed to quantify free viral RNA or intracellular viral RNA by quantitative real-time PCR (RT-qPCR), using the QIAmpViral RNA mini kit (Qiagen, Germantown, MD, USA) according to the manufacturer's instructions. Viral RNA copy counts/µL were calculated from calibration curves based on standards provided by the manufacturer (Genesig, Germantown, MD, USA).

### 2.9 Confocal microscopy

Vero cells were treated with compounds (10 µM) in a µ-Slide 8 Well (IbidiGmbH, Gräfelfing, Germany) in the dark for 60 min at 37 °C. DMSO-treated cells were used as negative controls. The samples were analyzed for fluorescent signal distribution and intensity using a Leica SP8 confocal microscope (Leica, Germany) equipped with a white laser. Confocal microscopy images were acquired using a Leica HCX PL APO 63 × objective. The excitation laser was set to 488 nm, and the hybrid emission detector (HyD) was set to 500–540 nm.

### 2.10 Spectrofluorometric analysis of perylene compounds and their interaction with liposomes

The samples’ steady-state fluorescence characteristics were measured in L-format using a Chronos DFD Fluorescence spectrometer (ISS, USA) equipped with a 300 W Cermax xenon arc lamp (ISS, USA), a concave holographic grating monochromator, and a PMT detector. The required amount of each sample was diluted in DMSO and measured in a 1-cm quartz cuvette at a constant temperature of 25 °C. The resulting data were evaluated using Vinci software (ISS, USA) and correlated to the utilized optical configuration.

Absorption spectra were measured using a high-performance Specord S 600 diode spectrophotometer (Jena Analyst, DE). The required amount of each sample was diluted in DMSO, added to 1-cm quartz cuvette, and measured within the selected absorbance spectrum range, against pure DMSO as a blank, at a constant temperature of 25 °C. The results were evaluated using WinASPECT® software (Jena Analyst, DE).

Fluorescence quantum yield was determined following a previously described protocol (Kozma et al., 2005; Magde et al., 1999; Williams et al., 1983). The kinetics of the incorporation of compounds into liposome membrane models was determined using steady-state fluorescence spectroscopy at a constant excitation and emission wavelength, according to the corresponding sample excitation and emission maxima. We added 50 μL of LNP suspension to 0.1 mM of the analyte in PBS, and monitored the increase of fluorescence intensity over the time range from 0 to 1400s. The preparation and characterization of the liposomal suspension is described in the supporting information (Fig. S4).

### 2.11 Quantification of cellular stress marker expression

RT-qPCR was performed to evaluate changes in the gene expression of cellular stress markers. CaCo-2 cells were cultivated in 24-well plates and exposed to the tested compound **13** (10 µM) for 5, 24, and 48 h. The cells were simultaneously treated with 2 mM KBrO_3_ (Penta, Czech Republic), 100 nM THA (thapsigargin; Sigma-Aldrich, Missouri, USA), and 10 nM TCDD (Cambridge Isotope Laboratories, Massachusetts, USA) as model cellular stress inducers. Previous publications report the primer sequences and numbers of the Universal Probe Library (UPL; Roche Life Sciences, Germany) used for detection of ATF3, CDKN1A (p21), CYP1A1, DDIT3, HMOX1, HSPA1B (HSP70), and HSPA5 mRNAs (Procházková et al., 2018; Šimečková et al., 2020). The TXNRD1 mRNA (NM_003330.2) level was detected using the forward primer, acacaaagcttcagcatgtca; reverse primer, caattccgagagcgttcc; and UPL probe #81. TaqMan™ Gene Expression Assays (Thermo Fisher Scientific, Waltham, MA, USA) were used to determine the levels of FGF21 (Hs00173927_m1), GADD45A (Hs00169255_m1), GDF15 (Hs00171132_m1), and SRXN1 (Hs00607800_m1). The amplification was run in a 10-µL reaction mixture containing 1 µL of sample, using Kapa Probe Fast One-Step (Kapa Biosystems Pty, Cape Town, South Africa) on a LightCycler® 480 System (Roche Diagnostics, Czech Republic) using the previously published sequence (Procházková et al., 2018). Reference gene qPCR assays (Generi Biotech, Hradec Králové, Czech Republic) were used for the following housekeeping genes: human β2-Microglobulin (B2M; #3030), hydroxymethylbilane synthase (HMBS; #3032), and RNA polymerase II subunit A (POLR2A; #3035). We used an average of three reference gene Cp values to calculate the change in gene expression using the comparative threshold cycle method (Schmittgen and Livak, 2008).

### 2.12 Rate of reactive oxygen (ROS) measurement

The ROS generation rate was estimated using the spectrophotometric method, based on the absorption changes of 1,3-diphenylisobenzofuran (DPBF, Sigma Aldrich, Germany) in methanol solution. Irreversible oxidation of DPBF by singlet oxygen results in peroxide formation, and DPBF bleaching. When light of 400–450 nm was used as actinic, DPBF bleaching occurred without any photosensitizer (PS) in solution. Therefore, to study photosensitized ROS generation, we used a white MCWHLP1 LED (Thorlabs, USA) with filters to limit the radiation to the 450–470 nm range (5.5 mW/cm^2^).

Spectrophotometric measurements were performed in a Qpod 2e (Quantum Northwest, USA) thermostated cuvette holder at 25 °C with magnetic stirring (500 rpm). Absorption spectra were recorded using a MayaPro spectrophotometer (Ocean Optics, USA) and a stabilized white light source with a SLS201L tungsten lamp (Thorlabs, USA). Illumination was uniform over the entire volume of the cuvette, to prevent artifacts associated with the diffusion of non-reacted components into the illuminated volume of the cuvette. In this case, the rate of irreversible bleaching of DPBF (412 nm) was the same under both continuous and pulsed irradiation with equal total irradiation time. Illumination was performed in a pulsed mode (Fig. S5A), with 1 s of illumination followed by 5 s of dark adaptation, during which the absorption spectrum of the PS-DPBF solution was recorded. Data processing was performed as previously described (Gvozdev et al., 2020).

ROS generation quantum yield for compounds **1**–**20** in methanol was calculated according to the equation

(1) $$\varphi_{ROS}=\varphi_{ROS}^{0}*\frac{r}{r^{0}}\frac{\left(1-10^{-A^{0}}\right)}{\left(1-10^{-A}\right)},$$

where r is the rate of DPBF photobleaching in solution of the PS, A is PS absorbance in the illumination region, and index 0 refers to a reference PS (we used riboflavin with $\varphi_{ROS}^{0}=0.51$ in methanol (Sikorska et al., 2005)).

ROS generation was also estimated using 9,10-dimethylanthracene (DMA), another specific fluorescent singlet oxygen trap, which forms nonfluorescent 9,10-dimethylanthracene endoperoxide after ROS-mediated DMA oxidation. Compounds (0, 0.1, 1, and 10 µM) in DMSO were mixed with DMA (0.5 µM), and these solutions were irradiated for 10 min at room temperature (RT) with light-emitting diodes (LED, 465–480 nm) having approximate power density of 30 mW/cm^2^ (device description in Fig. S7). DMA fluorescence intensity was measured at ex/em wavelengths of 370/432 nm in the presence or absence of α-tocopherol (3 µg/mL) as an antioxidant. Singlet oxygen generation was accompanied by a decreased fluorescence signal of the DMA probe (Zeng et al., 2020).

### 2.13 Studies of photodynamic inactivation of SARS-CoV-2 virions

Virus in DMEM (titer of 10^5^ PFU/mL) was mixed with the compounds (0–10 µM) in a microtiter plate, in triplicate, and irradiated for 10 min at RT with LEDs (465–480 nm) having an approximate power density of 30 mW/cm^2^ (device description in supplement). As a negative control, virus was incubated for 10 min in the dark at RT. Subsequently, both irradiated and non-irradiated virus samples were incubated in the dark at 37 °C for 60 min. All manipulation with the samples before and after this incubation, i.e., mixing the compound with the virus, pipetting into a microtiter plate, etc., were performed in daylight. Viral titers were determined by plaque assays (performed also in daylight) and expressed as PFU/mL, as previously described (Stefanik et al., 2021). To eliminate the influence of daylight on compound activity, the whole experiment, including all manipulation with samples, was performed in a dark room under red light. The virus sample was mixed with the indicated compounds at concentrations from 0 to 10 μM or 100 μM, incubated for 60 min at 37 °C and virus viability was evaluated using a plaque assay (Fig. 9A).

### 2.14 Studies of light-induced cytotoxicity of perylene compounds

To determine the light-induced cytotoxicity of the tested compounds, Vero cells were cultured for 24 h in 96-well plates to form a confluent monolayer, and then were treated with the tested compounds at concentrations of 0 to 10 µM. Then, the compound-treated cells were irradiated for 10 min at RT with LEDs (465–480 nm) having an approximate power density of 30 mW/cm^2^ (device description in Fig. S7). As a negative control, compound-treated cells were incubated for 10 min in the dark at RT. Subsequently, both irradiated and non-irradiated cell monolayers were incubated in the dark at 37 °C for 24 h. Then, the cell culture medium was aspirated and the potential cytotoxicity of the tested perylene derivatives was determined based on cell viability using Cell Counting Kit-8 (Dojindo Molecular Technologies, Munich, Germany) according to the manufacturer's instructions.

### 2.15 Evaluation of perylene compound solubility in 15% DMSO

To determine the “solubility” in 15% DMSO (Proskurin et al., 2018), the compounds were dissolved in DMSO, followed by dilution with water to a 15% DMSO concentration (v/v), centrifugation (12,000 rpm, 10 min), dilution of the supernatant to 50% DMSO, and photometry (with further dilution using 50% DMSO). The following values were used for calculations: Compounds **1**–**13**: ε 41,000 L mol^−1^ cm^−1^ (λ_max_ 470 nm); Compound **15**: ε 38,000 L mol^−1^ cm^−1^ (λ_max_ 470 nm); Compound **14**: ε 27,000 L mol^−1^ cm^−1^ (λ_max_ 460 nm); and Compounds **16**–**19**: ε 21,000 L mol^−1^ cm^−1^ (λ_max_ 470 nm). Table S1 presents the solubility data.

### 2.16 Statistical data analysis

Data were analyzed using the Shapiro-Wilk normality test and *t*-test or one-way ANOVA followed by Turkey's multiple comparisons test. Statistical analyses were performed using GraphPad Prism 7.04 software (GraphPad Software, Inc.). Differences with *P* values of <0.05 were considered significant.

## 3 Results

### 3.1 Perylene compounds showed low or no cytotoxicity towards Vero, CaCo-2, or CRFK cells

We initially determined the cytotoxicity of all studied compounds in Vero cells incubated with the tested compounds at concentrations of 2 and 10 µM for 48 h. The vast majority of our *in vitro* studies were performed in Vero cells because this cell line is highly suitable for culturing and antiviral assays with SARS-CoV-2. Most of the tested perylene derivatives showed a good cytotoxicity profile with CC_50_ values > 10 µM (Fig. 2A), and did not cause any morphological changes or cell proliferation disorders at the studied concentrations (data not shown). Interestingly, Vero cells treated with compounds **5** and **20** (with the perylene-ethynyl scaffold substituted with small charged groups); **4, 8, 14**, and **19** (substituted with non-polar groups); and **12** (substituted with a hydroxylated hexylamide chain) showed moderately decreased cell viability at 10 µM, but the cell viability did not decline below the threshold of 75% with any compound. The selected compounds **2, 13**, and **17** were well-tolerated by two other cell lines, CaCo-2 (derived from human colon adenocarcinoma) and CRFK (feline kidney cortex cells), up to 10 µM (Fig. 4B and C, left panels), indicating that the studied perylene derivatives were non-cytotoxic or showed only a moderate cytotoxicity (at the highest concentrations tested) towards multiple cell lines of different origins.

### 3.2 Amphipathic perylene derivatives showed nano- or submicromolar anti-SARS-CoV-2 activity

**Fig. 3:**
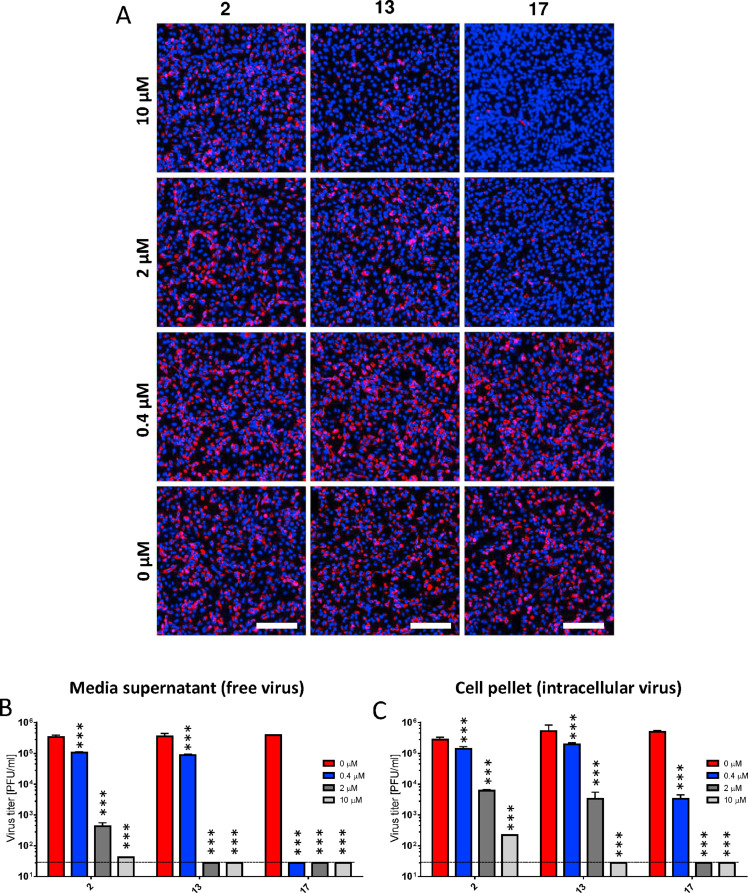
Suppression of coronaviral surface protein S1 expression, and inhibition of viral RNA synthesis, by compounds **2, 13**, and **17** at the indicated concentrations. (A) Immunofluorescence staining of Vero cells infected with SARS-CoV-2 (Wuhan variant) at a MOI of 0.1, and treated with the indicated compounds. At 48 h after infection, Vero cells were fixed on slides, stained with a rabbit coronavirus-specific primary antibody, and then with an Alexa Fluor 647-conjugated anti-rabbit goat secondary antibody (red), and finally counterstained with DAPI (blue). Scale bars, 200 μm. (B and C) After treatment with compounds **2, 13**, and **17** at the indicated concentrations, viral titers were determined from growth medium (media supernatant, B) and from infected cells (cell pellet, C). The cells were frozen twice and then thawed to rescue intracellular virions. Viral titers were quantified by plaque assays. Data are expressed as the mean ± SD of two independent experiments, each performed in triplicate. The horizontal dashed line indicates the minimum detectable threshold of 1.44 log_10_ PFU/mL. The means are significantly different from those of virus-infected DMSO-treated cells at ***P < 0.001. (For interpretation of the references to color in this figure legend, the reader is referred to the web version of this article.)

**Fig. 4:**
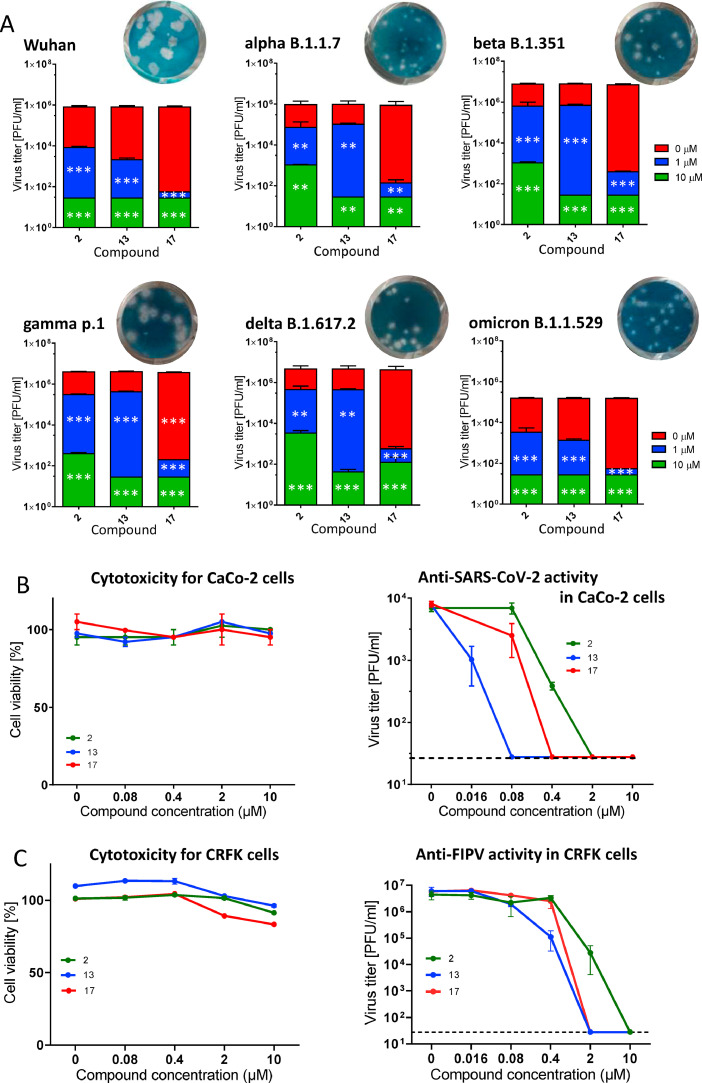
Antiviral activity and cytotoxicity of compounds **2, 3**, and **17**, determined for multiple SARS-CoV-2 variants and FIPV in Vero, CaCo-2, and CRFK cells. (A) Anti-SARS-CoV-2 activity of the compounds (at 0, 1, and 10 μM) against the indicated SARS-CoV-2 variants (Wuhan, Alpha B.1.1.7, Beta B.1.351, Gamma p.1, Delta B.1.617.2, and Omicron B.1.1.529). The characteristic plaque morphology of each viral strain is shown. (B) Cytotoxicity and anti-SARS-CoV-2 activity (Wuhan strain) of the indicated compounds at concentrations of 0–10 μM in CaCo-2 cells. (C) Cytotoxicity and antiviral activity of the indicated compounds in CRFK cells, with antiviral activity detected against feline infectious peritonitis coronavirus (FIPV). Antiviral assays were performed as described in Fig. 2B. Data are expressed as the mean ± SD of two independent experiments, each performed in triplicate. The horizontal dashed line indicates the minimum detectable threshold of 1.44 log_10_ PFU/mL. The means are significantly different from those of virus-infected DMSO-treated cells at **P < 0.01 and ***P < 0.001. (For interpretation of the references to color in this figure legend, the reader is referred to the web version of this article.)

Our SAR study was performed using compounds formed by a large planar perylene residue (a hydrophobic “tail”) and structurally divergent polar “heads”, which are connected to the perylene group by a rigid ethynyl or thiophene spacer (Fig. 1). The aim of this study was to elucidate how the anti-SARS-CoV-2 activity was influenced by the size, shape, rigidity, and polarity of the hydrophilic “heads” (i.e., structural characteristics affecting the overall compound amphipathicity). All compounds were initially tested for their antiviral effect in concentrations ranging from 0 to 10 µM, which were found to be non-cytotoxic towards Vero cells. Viral titer reduction assays demonstrated that most of the amphipathic perylene compounds inhibited the production of viable viral particles in virus-infected Vero cell culture, with a dose-dependent antiviral effect (Figs. 2C–F, 3B and C, and 4B and C). Selected compounds (**2, 13**, and **17**) were subjected to advanced studies of their biological properties. We demonstrated that these three derivatives also suppressed the synthesis of viral genomic RNA (Fig. S2) and were found to reduce the expression of a SARS-CoV-2 surface antigen (spike protein S1) (Fig. 3A). As these compounds are extremely sensitive to light, all antiviral assays were performed under stable/constant light conditions (flow box lights off, all incubations performed in the dark, etc.) to exclude unwanted changes in their antiviral activity due to compound photosensitization.

#### 3.2.1 Nucleoside-derived perylenylethynyl compounds

We first investigated the anti-SARS-CoV-2 activity of two well-described nucleoside-derived compounds: 5-(perylen-3-yl)ethynyl-*arabino*-uridine (aUY11) (**1**) and 5-(perylen-3-yl)ethynyl-2′-*deoxy*-uridine (dUY11) (**2**). These two derivatives share the same three-dimensional shape and rigidity, and differ only in the presence or absence of the 2′*C* hydroxyl on the hydrophilic (sugar) part of the molecule, and likely also in stereochemistry of the sugar ring. Both compounds exhibited similar antiviral potency against SARS-CoV-2 (Wuhan variant), with EC_50_ values of 0.406 µM for **1** and 0.256 µM for **2**, indicating that hydroxylation of the 2′-carbon of the ribose residue did not eliminate the virus-inhibitory activity (Fig. 2C). This observation also clearly indicates that the molecular mechanism of antiviral action for both molecules was not based on suppression of viral genomic RNA synthesis by highly specific and selective interaction with viral RNA-dependent RNA polymerase (RdRp), which is the typical mechanism of virus inhibition by most nucleoside analogues.

#### 3.2.2 Aglycosylated (ribose-free) perylenylethynyl compounds

We further evaluated the anti-SARS-CoV-2 activity of two aglycosylated (ribose-free) non-nucleoside perylene derivatives (aglycones **3** and **4**). Surprisingly, compound **3** exerted low antiviral potency against SARS-CoV-2 (EC_50_ value > 2.5 µM). In contrast, compound **4**, which is a methylated derivative of **3**, exerted sub-micromolar anti-SARS-CoV-2 activity (EC_50_ of 0.406 µM) (Fig. 2C).

#### 3.2.3 Perylenylethynyluracil compounds with pentose sugar replacement

Compounds **5**–**13** are perylenylethynyluracil compounds in which the pentose sugar is replaced with structurally divergent moieties substituted at the *N1* position of the uracil residue. These structures possess a lipophilic pivaloyloxymethyl (Pom) group at the *N3* position to increase the solubility of the poorly soluble perylenylethynyluracil scaffold. We observed that *N1* substituents replacing/mimicking the sugar ring had different effects on anti-SARS-CoV-2 activity, depending on their size, polarity, and rigidity. The introduction of a small highly polar group (carboxymethyl in compound **5**) or a highly hydrophobic group (buthylamide in compound **8**) resulted in significantly reduced antiviral potency (EC_50_ of ∼2 µM). On the other hand, compounds with ɷ-hydroxylated alkylamide groups of various length (compounds **6** and **9**–**11**) showed sub-micromolar antiviral activities (EC_50_ of 0.139–0.432 µM) (Fig. 2D). Interestingly, compound **12**, which has the longest and most flexible alkylamide chain of all tested compounds, also showed lower anti-SARS-CoV-2 potency (EC_50_ of 0.812 µM). Perylene derivatives with expanded and more hydrophilic dihydroxyalkylamide groups (compounds **7** and **13**) exhibited EC_50_ values similar to those of compounds **6** and **9**–**11**. Moreover, at higher concentrations (2 and 10 µM), these derivatives caused complete inhibition of SARS-CoV-2 replication in virus-infected cells. Our results clearly indicated that the ribose residue was not necessary for the anti-SARS-CoV-2 activity of perylenylethynyluracils and could be successfully replaced with various structurally divergent moieties. Moreover, it was evident that high anti-SARS-CoV-2 efficacy required a balance between the hydrophobic and hydrophilic parts of the amphipathic perylene molecule; both the most hydrophilic and most hydrophobic compounds showed no/low activity, whereas derivatives having medium polarity showed the best antiviral profiles. These properties may be related to the compounds’ solubility in water and/or their ability to interact with the viral membrane.

#### 3.2.4 Phenylethynylperylene compounds (without sugar and with uracil replaced with a p-substituted phenyl (aniline) group)

Next, we analyzed the anti-SARS-CoV-2 activity of two perylenylethynyl anilines (compounds **14** and **15**), in which the uracil unit was replaced with aniline-derived groups of various polarity. The *N*-acetyl aniline derivative **15** exerted two-digit nano-molar anti-SARS-CoV-2 activity (EC_50_ of 0.063 µM) (Fig. 2E). Thus, replacement of the whole ribose-uracil part of the perylenylethynyluracil scaffold could generate compounds with very strong anti-SARS-CoV-2 activity. In contrast, substitution with a more hydrophobic *N,N*-dimethyl aniline in compound **14** resulted in complete loss of antiviral potency (EC_50_ > 2.5 µM) (Fig. 2E). Thus, eliminating amphipathicity by introducing a highly hydrophobic substituent into the perylenylethynyl scaffold resulted in a substantial decrease in anti-SARS-CoV-2 efficacy. Moreover, the weak anti-SARS-CoV-2 activity of compound **14** is related to its poor ability to generate ^1^O_2_, as described below.

#### 3.2.5 Perylenylthienyl compounds (with sugar-uracil-ethynyl replacement)

The last tested group of amphipathic perylene compounds included thiophene-containing compounds lacking the rigid ethynyl linker. The thiophene-containing compounds **16**–**19** showed structure-activity relationship trends similar to those observed for the perylenylethynyluracil derivatives **5, 6, 8**, and **13**, because both of these compound families have similar structural features in the hydrophilic parts (“heads”) of the molecule. The thiophene-containing compounds showed the following antiviral potencies, in increasing order: **16** (with a small highly polar carboxyl group), EC_50_ of 0.536 µM; **19** (with a non-polar buthylamide group), EC_50_ of 0.098 µM; **18** (with two hydroxyethylamide chains), EC_50_ of 0.019 µM; **17** (with a ɷ-hydroxylated buthylamide group), EC_50_ of < 0.004 µM (Fig. 2F). Finally, the simplest tested compound, **20** (containing only a hydrophobic perylene core with a polar carboxyl group), showed no/negligible antiviral potency against SARS-CoV-2, with an EC_50_ > 2.5 µM (Fig. 2F). Based on these results, we can conclude that the rigid ethynyl linker is not critical for maintaining the anti-SARS-CoV-2 potency of perylene compounds. In fact, the antiviral activity of some perylene-based derivatives increased more than 10-fold when the ethynyl linker was replaced by a thiophene bridge. Among the whole tested series, compound **17** was the most potent inhibitor of SARS-CoV-2 in Vero cells. The increased antiviral activity of thiophene-containing compounds was likely related to their superior ability to generate ^1^O_2_, as shown below.

### 3.3 Amphipathic perylene compounds showed antiviral potency against multiple coronaviruses in various cell lines

All of the above-described antiviral assays were performed using the original Wuhan variant of SARS-CoV-2. We further investigated the ability of selected perylene derivatives (**2, 13**, and **17**) to suppress the replication of multiple SARS-CoV-2 variants, which were isolated from patients in the Czech Republic during 2020–2022. SARS-CoV-2 variants have differing capacities for replication in Vero cell culture, as manifested by differences in plaque morphology (Fig. 4A). However, the replication of all tested SARS-CoV-2 variants was efficiently inhibited by the studied compounds, when applied at concentrations of 1 and 10 µM and cultivated for 48 h at 37 °C under 5% CO_2_ (Fig. 4A). We further demonstrated that the selected compounds showed sub-micromolar anti-SARS-CoV-2 activity in CaCo-2 cells (Fig. 4B) as well as potently suppressed the replication of FIPV, a causative agent of feline infectious peritonitis of domestic cats and wild felines, in CRFK cells (Fig. 4C). Thus, amphipathic perylene compounds were able to suppress replication of multiple members of the *Coronaviridae* family, and the perylene-mediated antiviral effect was not dependent on cell type.

### 3.4 Amphipathic perylene compounds target the SARS-CoV-2 envelope and block the virus-cell fusion process

Our anti-SARS-CoV-2 assays revealed that the perylene compounds greatly reduced the titer of free virus in the media supernatant (Figs. 2C-G, 3B). However, the inhibitory effect of the perylene compounds was significantly weaker when we determined the titer of virus replicating in host cells (intracellular virus) (Fig. 3C) or when we monitored the expression of viral spike protein (surface antigen) in the infected cell culture by immunofluorescence staining (Fig. 3A). This observation suggests that perylene compounds act directly on SARS-CoV-2 particles in the early stages of infection (free virus), but have a limited inhibitory effect on virus that is already replicating in host cells (intracellular virus). Since such activity has typically been described for several photosensitizing inhibitors of virus-cell fusion, particularly porphyrins (Assunção-Miranda et al., 2016), we focused on studying the mechanism of antiviral action of perylene compounds in detail.

**Fig. 5:**
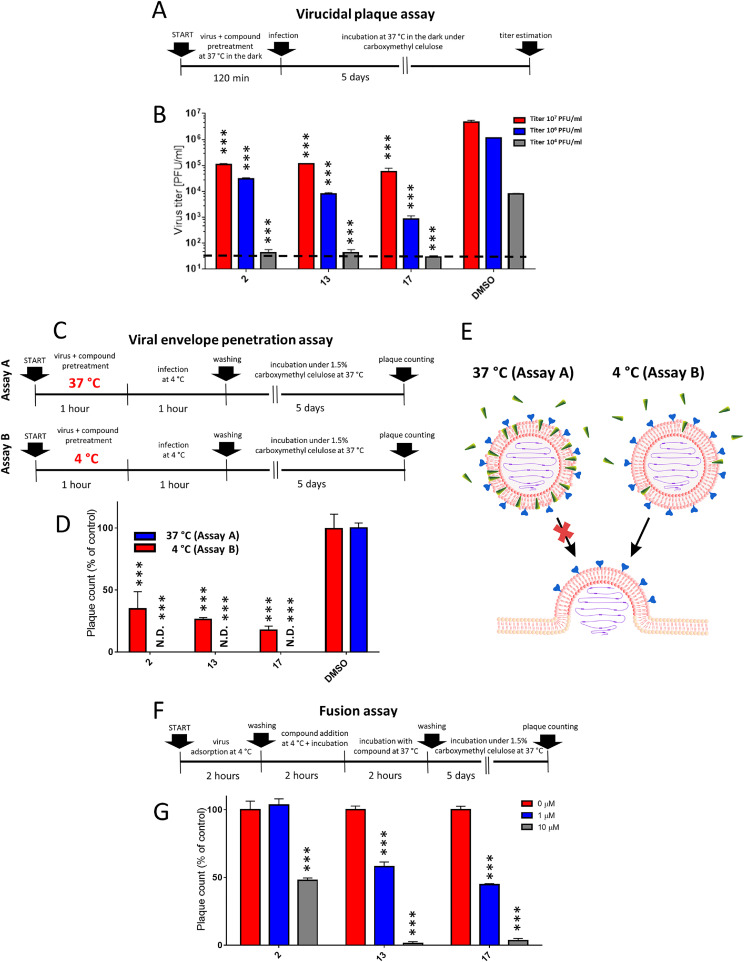
Mechanistic studies of the antiviral effects of compounds **2, 13**, and **17**. (A and B) Demonstration of the direct (virucidal) activity of compounds against SARS-CoV-2 in Vero cells. (A) Schematic representation of the experiment. (B) Quantification of the virucidal activity of the compounds in Vero cells. The virus (at titers of 10^4^, 10^6^, and 10^7^ PFU/mL) was incubated with the compounds (10 μM) for 120 min. Viral titers were then quantified by plaque assays. (C–G) Mechanistic studies of the interaction of selected compounds with the SARS-CoV-2 envelope membrane and blocking of the virus-cell fusion process. (C) Schematic representation of a viral envelope penetration assay. (D) Quantification of virus infectivity after pre-incubation of the virus with the compounds for 1 h at different temperatures: 37 °C (assay A) and 4 °C (assay B). (E) Schematic representation of the virus–cell fusion process after incubation of the virus with compounds at 37 °C and 4 °C. (F) Inhibition of virus-cell fusion with the indicated compounds (a fusion assay, schematic representation). (G) Quantification of virus infectivity using the fusion assay. The virus was attached to the cell surface at 4 °C (but not fused), then the compounds were added, and the temperature was raised to 37 °C to allow fusion. Data are expressed as the mean ± SD of two independent experiments, each performed in triplicate. The horizontal dashed line indicates the minimum detectable threshold of 1.44 log_10_ PFU/mL. The means are significantly different from those of virus-infected DMSO-treated cells at ***P < 0.001. (For interpretation of the references to color in this figure legend, the reader is referred to the web version of this article.)

To study the molecular mechanism underlying the anti-SARS-CoV-2 action of perylene compounds in greater detail, we first investigated whether perylene derivatives exerted direct (virucidal) activity against SARS-CoV-2, i.e., whether the compounds inactivated SARS-CoV-2 virions before the virus entered the host cell. SARS-CoV-2 (Wuhan variant) at different initial titers (10^4^, 10^6^, or 10^7^ PFU/mL) was pre-treated with the selected compounds (**2, 13**, or **17**) for 120 min at 37 °C in the dark, and then the virus viability was determined using a plaque assay (Fig. 5A). After 5 days of incubation at 37 °C and 5% CO_2_, under 1.5% carboxymethylcellulose, we observed significantly decreased viral titers in the compound-treated virus samples (10^1^ to 10^3^-fold, depending on the initial virus titer) (Fig. 5B). Among the tested derivatives, compound **17** exerted the strongest virucidal effect against SARS-CoV-2. Our results indicated that perylene derivatives exerted a direct virus-damaging effect at the very beginning of viral infection and prevented SARS-CoV-2 from entering the host cell.

To demonstrate the interaction of perylene compounds with the SARS-CoV-2 envelope, we performed another experiment based on the compound–virus interaction at a low temperature. We hypothesized that if the perylene derivatives were incorporated into the viral membrane, a low temperature would be associated with lower fluidity of the viral membrane, and consequently with less efficient membrane incorporation of the perylene compounds, and ultimately a lower antiviral effect. This phenomenon was studied with selected perylene derivatives (**2, 13**, and **17**), using specialized cell-based assays. First, the virus was pre-incubated with the compounds (10 µM) at two different temperatures (37 °C Fig. 5C, assay A; or 4 °C, Fig. 5C, assay B). Next, Vero cells were infected with the virus at 4 °C, the unadsorbed virus was washed off, and the virus-infected cells were cultivated under 1.5% carboxymethyl cellulose for 5 days at 37 °C and 5% CO_2_ (Fig. 5C). The results showed that virus pre-treatment with the tested compounds at 37 °C (assay A, normal membrane fluidity) completely inhibited the virus’ ability to form plaques on the Vero cell monolayer (high antiviral activity). On the other hand, after virus pre-treatment with the compounds at 4 °C (assay B, lower membrane fluidity), the virus’ viability was partially restored (the virus formed plaques on Vero monolayers, indicating lower antiviral activity) (Fig. 5D). This findings suggested that envelope fluidity was required for the perylene compounds' virus-inhibitory activity, as schematically represented in Fig. 5E.

Finally, we performed a cell-based fusion assay to prove our hypothesis that perylene compounds act as blockers of the virus-cell fusion machinery. In this assay, the virus was allowed to attach to the host cell surface at 4 °C, but fusion was blocked due to the low temperature. After washing out the unadsorbed virus and adding compounds **2, 13,** or **17** (at 0, 1, and 10 µM), the temperature was raised to 37 °C to allow fusion to occur. Then, the infected cells were cultured under 1.5% carboxymethyl cellulose for 5 days (Fig. 5F). We hypothesize that if the compounds block virus-cell fusion, we will observe a lower number of plaques in virus-infected cells treated with the compounds than in controls. In fact, we observed a statistically significant reduction in plaque numbers in the infected cells treated with compounds **13** and **17** at both concentrations tested (note: compound **17** caused observable cytotoxicity to Vero cells at 10 µM, but plaques were still countable). Interestingly, compound **2** significantly decreased plaque counts only at 10 µM (not at 1 µM) compared to control cells (Fig. G). Our observations support our hypothesis that perylene compounds targeted the SARS-CoV-2 envelope and blocked the virus–cell fusion machinery. This effect will be discussed below in relation to the compounds’ photosensitization and ability to generate ^1^O_2_.

### 3.5 Amphipathic perylene derivatives target cellular membranes

**Fig. 6:**
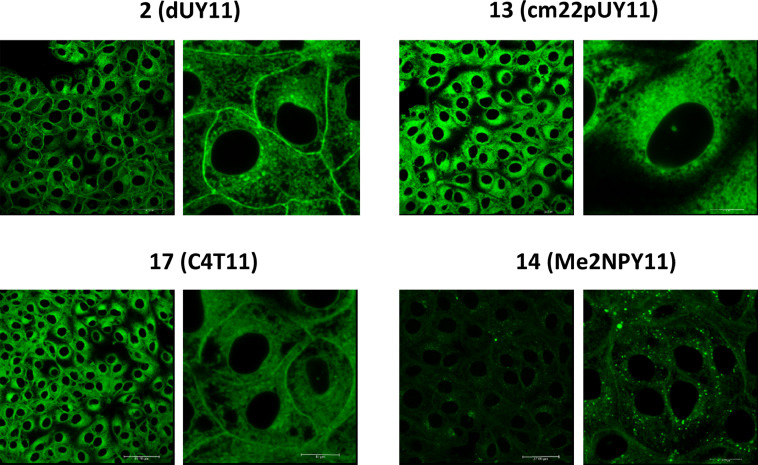
Penetration of compounds **2, 13, 14**, and **17** into Vero cells. Compound **14** was used as a negative control. Cells were seeded on slides for 24 h, then treated with the compounds at a concentration of 10 µM and incubated for 1 h. Photomicrographs were taken using confocal microscopy. (For interpretation of the references to color in this figure legend, the reader is referred to the web version of this article.)

To investigate the interaction of perylene compounds with cellular membranes, we took advantage of their intrinsic fluorescence, which is typical for perylene compounds after their incorporation into lipid bilayers. For this experiment, we selected compounds **2, 13**, and **17** (having high anti-SARS-CoV-2 activity) and compound **14** (showing no antiviral effect) as a negative control. Confocal microscopy revealed that perylene chromophores at a concentration of 10 µM were extensively incorporated into Vero cell membranes (Fig. 6). This was not surprising, since viral envelopes are derived from cellular membranes, such that both share similar structural and biophysical properties. Compounds **2** and **17** were predominantly incorporated into the cytoplasmatic membrane and the nuclear envelope, whereas compound **13** was mainly localized in intracellular membranes and nuclear envelopes, and even in the nuclei. Surprisingly, compound **14** preferentially accumulated inside the cytoplasmic vesicles, likely endosomes or lysosomes. The fluorescence signal of membrane-integrated compound **14** was relatively weak (Fig. 6), possibly due to the low affinity of **14** for membranes, the low fluorescence quantum yield (8%) determined for **14** (Fig. S3C), or the low solubility of **14** in aqueous solutions due to its high hydrophobicity.

### 3.6 Amphipathic perylene derivatives intercalate into liposomal lipid bilayers

We further explored whether perylene compounds were incorporated into liposomes, which represent a protein-free lipid membrane model system of defined lipid composition. For this experiment, we selected compounds **13** and **16** as representative perylene derivatives with good anti-SARS-CoV-2 profiles (EC_50_ of 0.2735 and 0.5367 µM, respectively). We compared their fluorescence spectra with that of compound **20**, which showed no/negligible antiviral effect (EC_50_ > 2.5 µM) as a negative control. All compounds were tested at a concentration of 10 µM.

**Fig. 7:**
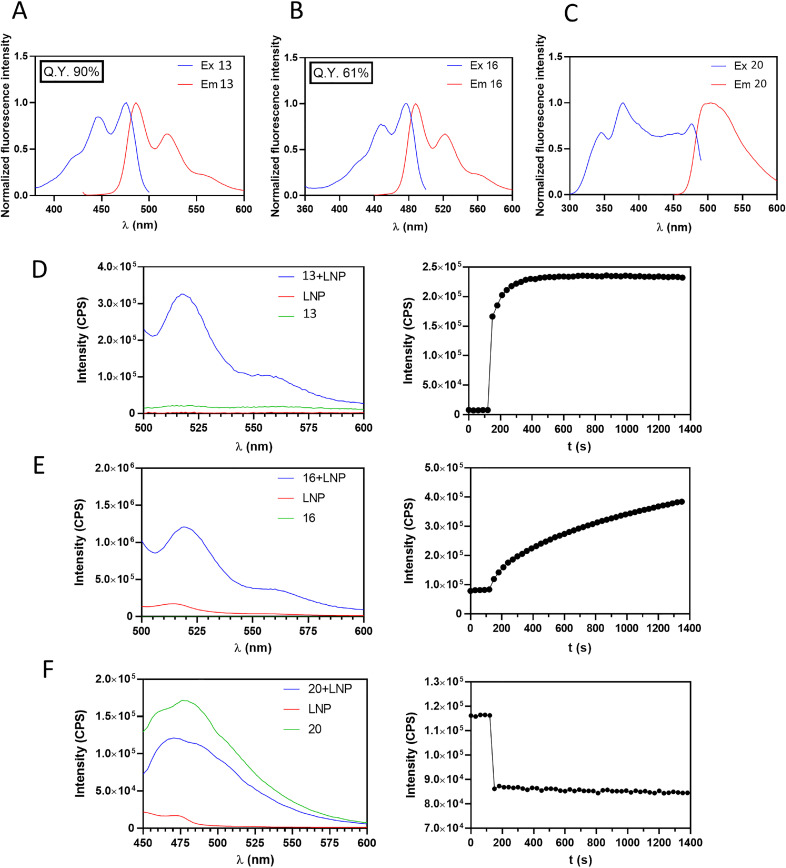
Fluorescence spectra and penetration of selected compounds **13, 16**, and **20** in liposomes. (A–C) Ex/Em spectra of the compounds in DMSO with the determined fluorescence quantum yields. (D–F) Fluorescence spectra and kinetics of penetration of compound **13** (D), compound **16** (E), and compound **20** (F). Left panels: Fluorescence spectra of free compounds in PBS (10 µM, green lines), free liposomes (LNP, red lines), and a mixture of a compound and LNP in PBS (blue lines). Right panels: Kinetics of the penetration of compounds (10 µM) into liposomes, measured at 520 nm for compounds **13** and **16**, and at 480 nm for compound **20**. (For interpretation of the references to color in this figure legend, the reader is referred to the web version of this article.)

Compounds **13** and **16** yielded very poor fluorescence signals when resolved in PBS. However, after the addition of unilamellar liposomes (EPC/Chol of 70/30 mol%), both compounds exhibited significant fluorescence activity with an emission maxima around 520 nm (Fig. 7D and E, left panel). These changes of fluorescence properties indicated that these perylene compounds penetrated the lipid bilayers. The kinetics of liposome penetration differed somewhat between compounds **13** and **16**; however, in general, both derivatives penetrated the liposomal membranes very quickly and efficiently. After the complete incubation period (1400s), the steady-state fluorescence signals (at 520 nm) reached similar intensities of about 10^5^ CPS (Fig. 7D and E, right panel). Notably, the high ability of compounds **13** and **16** to penetrate the liposomal membranes correlates well with their high antiviral activity (Fig. 2D and F, and Table 1). In contrast, compound **20** produced a strong fluorescence signal when dissolved in PBS (good solubility in water), but exhibited no fluorescence enhancement after mixing with liposomes (Fig. 7F). This indicates that **20** had poor affinity for membranes (low membrane penetration), which strongly correlates with its low/negligible anti-SARS-CoV-2 potency.

To elucidate the affinity of perylene derivatives for liposomal membranes in greater detail, we investigated the membrane penetration of several other compounds with generally lower anti-SARS-CoV-2 activity—namely compounds **8** (EC_50_ of 2.048 µM), **12** (EC_50_ of 0.8125 µM), and **14** (EC_50_ > 2.5 µM). We assumed that these compounds had low affinity for membranes, and that they therefore incorporated into liposomes with low efficiency. Indeed, compared to compounds **13** and **16**, compound **14** (with the lowest antiviral potency of the three derivatives studied) produced a fluorescence spectrum of a somewhat different shape, and significantly lower intensity, in the emission maximum (540 nm). Similarly, for compound **14**, the kinetics of liposome penetration were slow and very gradual, being characterized by relatively low fluorescence intensity at 540 nm (only 10^3^ CPS) (Fig. S3). This could indicate that **14** exhibits low affinity to liposomal membranes. However, it is also possible that the observed differences in fluorescence activity were related to the low quantum yield of **14** (8%), compared to those of **13** (90%) and **16** (61%) (Fig. 7A,B, and Fig. S3C). Additionally, **14** was poorly soluble in water (high hydrophobicity), which could also affect the degree to which the compound is incorporated into the membrane. Overall, the low compound–membrane interaction of **14** correlates well with its low anti-SARS-CoV-2 activity.

Interestingly, the behavior of the other two studied compounds, **8** and **12**, was very similar to the behavior of **13** and **16**—with all showing low fluorescence in PBS, increased fluorescence after mixing with liposomes, and similar kinetics of liposome penetration (Fig. S3). This is notable because compounds **8** and **12** differed significantly from **13** to **16** in terms of antiviral activity. This indicates that although effective penetration through the membrane was important, it was not the only condition required for strong anti-SARS-CoV activity of perylene derivatives.

### 3.7 Perylene compounds generate singlet oxygen under blue light irradiation

Earlier studies have suggested that amphipathic perylene compounds and other perylene-related structures can act as photosensitizers (Alferova et al., 2022; Mariewskaya et al., 2021; Vigant et al., 2014). Therefore, we studied in detail the ability of perylene derivatives to generate ROS (particularly ^1^O_2_) under irradiation with blue light (using a wavelength near the excitation maxima of perylene compounds). Initially, the ROS generation rate was spectrophotometrically analyzed as the absorption changes of a DPBF trap (^1^O_2_-mediated oxidation of DPBF trap results in DPBF bleaching). We determined the bleaching rate of DPBF in a solution with perylene compounds in methanol as the change in optical density at the absorption maximum of DPBF (412 nm), subtracting the contribution of perylene compound absorption, during the initial portion of the bleaching curve, normalized to irradiation time (Fig. S5). When the perylene derivatives were irradiated with blue light of 450–470 nm, we observed rapid bleaching of the DPBF trap. At the same time, the perylene compounds exhibited excellent photostability (over 99%). Values of ROS yield for compounds **1**–**20** are shown in Table 2. It was obvious that compounds **16–19** of the T11 series exhibited higher photodynamic activity than compounds **1–13** of the UY11 series, **14**–**15** of the NPY11 series and control compound **20**. The photodynamic activity of compounds **14** and **20** was minimal, which is in agreement with their low antiviral potency. The increased photodynamic activity of compounds **16–19** correlated with their extremely strong anti-SARS-CoV-2 potency, particularly for compounds **17** and **18**. Notably, compounds **1–15** were characterized by the presence of an absorption band in the 460–490 nm range, which should increase the efficiency of light absorption in the blue-green region of the spectrum. In order to reveal the involvement of this band in the photosensitization process, we compared the rate of the DPBF bleaching in solutions of compounds **1**–**20** under illumination in a wide range of wavelengths (450–600 nm). We have shown that the bleaching rate of DPBF correlates negatively with PS integrated optical density in the blue-green region. Thus, the long-wavelength absorption band of compounds **1**–**15** does not participate in the activation of triplet excited states.

**Table 2:** ROS generation quantum yields for compounds **1**–**20** in methanol.

Compound	$\varphi_{ROS}$,%
1	22.8 ± 0.4
2	20.9 ± 0.2
3	12.7 ± 1.2
4	22.8 ± 0.4
5	20.5 ± 0.2
6	18.2 ± 0.2
7	17.3 ± 0.2
8	17.6 ± 0.4
9	17.3 ± 0.2
10	16.5 ± 0.2
11	17.2 ± 0.4
12	18.1 ± 0.4
13	19.1 ± 0.2
14	7.1 ± 0.2
15	23.1 ± 0.4
16	59.0 ± 1.8
17	59.0 ± 2.4
18	60.0 ± 2.4
19	59.1 ± 1.2
20	20.0 ± 1.2

**Fig. 8:**
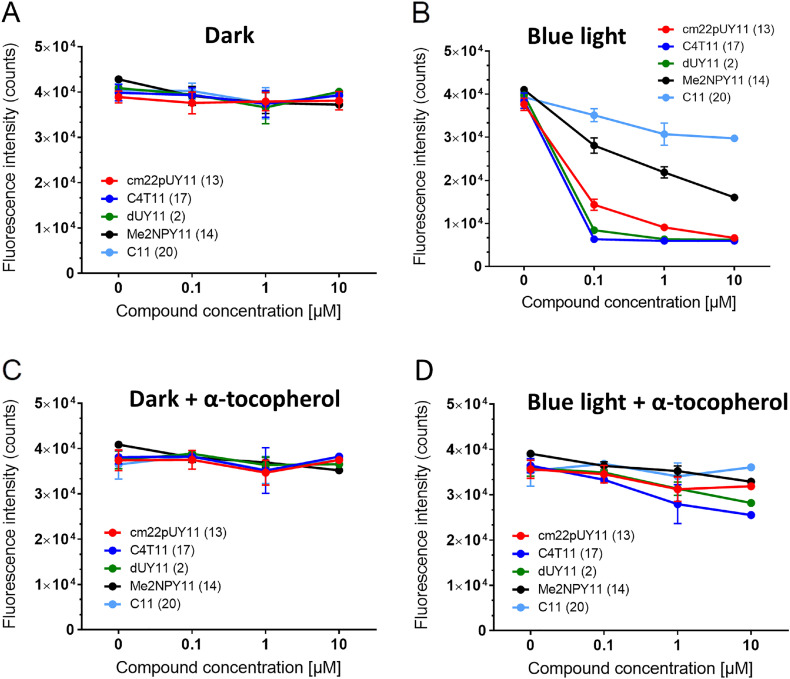
ROS generation measurement in the presence of DMA in DMSO. Selected compounds (**2, 13, 14, 17**, and **20**) were mixed with DMA (0.5 µM) and incubated for 10 min in the dark (A), under blue light (465–480 nm) (B), in the dark in the presence of α-tocopherol (C), and under blue light in the presence of α-tocopherol (D). Fluorescence intensity was then measured at ex/em of 370/432 nm. (For interpretation of the references to color in this figure legend, the reader is referred to the web version of this article.)

We further analyzed ROS generation using DMA, another specific fluorescent ROS trap that forms nonfluorescent DMA-endoperoxide in the presence of ^1^O_2_. Compounds **2, 13**, and **17** (all strong SARS-CoV-2 inhibitors) generated ROS in DMSO when irradiated by blue light (465–480 nm/30 mW/cm^2^), as indicated by significant bleaching of DMA trap fluorescence. In contrast, no/limited bleaching of the fluorescence signal was measured after incubation of the compounds with DMA in the dark or in the presence of α-tocopherol (an antioxidant). As expected, compounds **14** and **20** (negative controls) showed minimal photodynamic activities (Fig. 8).

### 3.8 Antiviral activity of perylene compounds is induced by blue light

**Fig. 9:**
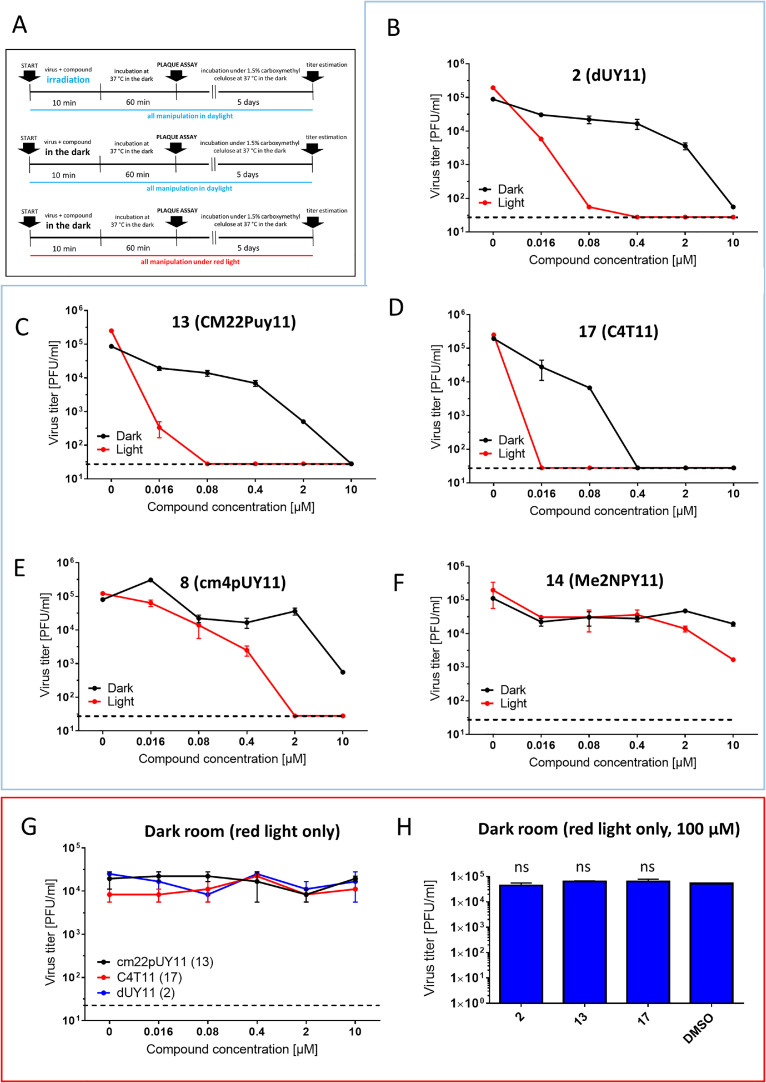
Light-dependent anti-SARS-CoV-2 activity of perylene compounds. (A) Schematic representation of the experiment. (B–F, blue box) Selected compounds **2** (B), **13** (C), **17** (D), **8** (E), and **14** (F) at the indicated concentrations were mixed with the virus (10^4^–10^5^ PFU/mL), irradiated for 10 min with blue light (465–480 nm) at RT, and then incubated for 60 min in the dark at 37 °C (red curves). The negative control was a virus–compound mixture incubated only in the dark (black curves). All manipulation with the samples before and after this incubation, i.e., mixing the compound with the virus, pipetting into a microtiter plate, etc., were performed in daylight. After incubation, virus viability was evaluated using a plaque assay (performed also in daylight). (G and H, red box) To eliminate the influence of daylight on compound activity, the experiment, including all manipulation with samples, was performed in a dark room under only red light. The virus sample was mixed with the indicated compounds at concentrations from 0 to 10 μM (G) or 100 μM (H), and then incubated for 60 min in the dark at 37 °C (no light irradiation). Finally, virus viability was evaluated using a plaque assay. Data are expressed as the mean ± SD of two independent experiments, each performed in triplicate. The horizontal dashed line indicates the minimum detectable threshold of 1.44 log_10_ PFU/mL. (For interpretation of the references to color in this figure legend, the reader is referred to the web version of this article.)

Since the studied compounds induced ROS formation under blue light irradiation, we next irradiated SARS-CoV-2 samples (titers of 10^4^–10^5^ PFU/mL) mixed with selected perylene derivatives **2, 13**, and **17** (and **8** and **14** as controls) with blue light (465–480 nm/30 mW/cm^2^) for 10 min, and then incubated the virus–compound mixture at 37 °C under 5% CO_2_ in the dark for 60 min. For negative controls, we used appropriate non-irradiated virus-compound mixtures (incubated for 10 min in the dark and then incubated at 37 °C under 5% CO_2_ in the dark for another 60 min). Note: all manipulation with both samples, i.e., mixing the compound with the virus, pipetting into a microtiter plate, plaque assays, etc., were performed in daylight. The viability of the compound-treated virus was subsequently examined by a plaque assay. To completely eliminate the influence of daylight on compound activity, we performed a parallel experiment in a dark room using only red light (light of 624 ± 20 nm was used as a work light) (Fig. 9A).

Irradiation of the virus-compound mixture with blue light significantly enhanced the antiviral activities of **2, 13**, and **17**, and shifted their inhibitory effects to low-nanomolar concentrations (Fig. 9B–D, red lines) compared to non-irradiated samples (Fig. 9B-D, black lines). We also observed a significant increase of antiviral activity for compound **8**, which showed a weak activity under dark conditions (Fig. 9E). Only a slight increase of antiviral potency was observed for compound **14** (viral titer decreased by less than an order of magnitude) (Fig. 9F).

Interestingly, the antiviral activity of compounds **2, 13,** and **17** (at concentrations of up to 10 µM) completely disappeared when the experiment was performed in a dark room under red light only (in the absence of daylight) (Fig. 9G). Even when applied at a 10-fold higher concentration (100 µM), the compounds showed no antiviral effect under red light (Fig. 9H). Finally, we can conclude that the perylene compounds lose their antiviral activity in the absence of daylight (Fig. 9G,H), and surprisingly, even a brief exposure of the compounds to daylight during sample preparation and pipetting of samples onto microtiter plates is sufficient to activate the photosensitizers and manifest their light-dependent antiviral activity, as shown in Fig. 9B-D (black lines).

In the next experiment, we examined the light-induced cytotoxicity of perylene compounds **2, 13,** and **17** (i.e., photosenzitisers with high antiviral activity) and compared them with **8** and **14** (controls). Irradiation with blue light for 10 min dramatically increased the cytotoxicity of compounds **2, 13,** and **17** (especially at concentrations of 0.4 to 10 µM) compared with the nonirradiated samples. On the other hand, blue light irradiation had no effect on the toxicity of **8** and **14** (Fig. S6). We can conclude that light-induced generation of ROS may have a significant effect on the cytotoxicity of photosenzitisers and that ROS -mediated oxidation of cellular targets may substantially affect cell metabolism, proliferation, and viability.

### 3.9 Perylene compounds do not induce expression of cellular stress markers under normal lighting conditions

To more deeply evaluate the cytotoxicity profile of perylene compounds for their potential use as pharmacophores, we next analyzed the expression of cellular stress markers in compound-treated CaCo-2 cells, when the experiment is performed under normal lighting conditions (in daylight) and the compounds are incubated with the cells in the dark. Cellular stress responses in cells are activated by various toxicants, and are detectable at the transcriptome level before visible manifestations of toxicity at the cellular level. Thus, the screening of genes activated by intracellular stress pathways may reveal mechanistic information about the biological effects of chemicals (Fulda et al., 2010; Limonciel et al., 2018; Simmons et al., 2009; Zhang et al., 2014). Our cellular stress response assays were performed using compound **13**, since it showed strong anti-SARS-CoV-2 activity in both Vero and CaCo-2 cells, and was also active against FIPV in CRFK cells. We mainly focused on markers of early cellular stress, oxidative stress, DNA damage response, endoplasmic reticulum (ER) stress, xenobiotic response, and heat shock response. Changes in target gene expression were determined by qRT-PCR in CaCo-2 cells exposed to compound **13** (at 10 µM) for 5 h, 24 h, and 48 h.

**Fig. 10:**
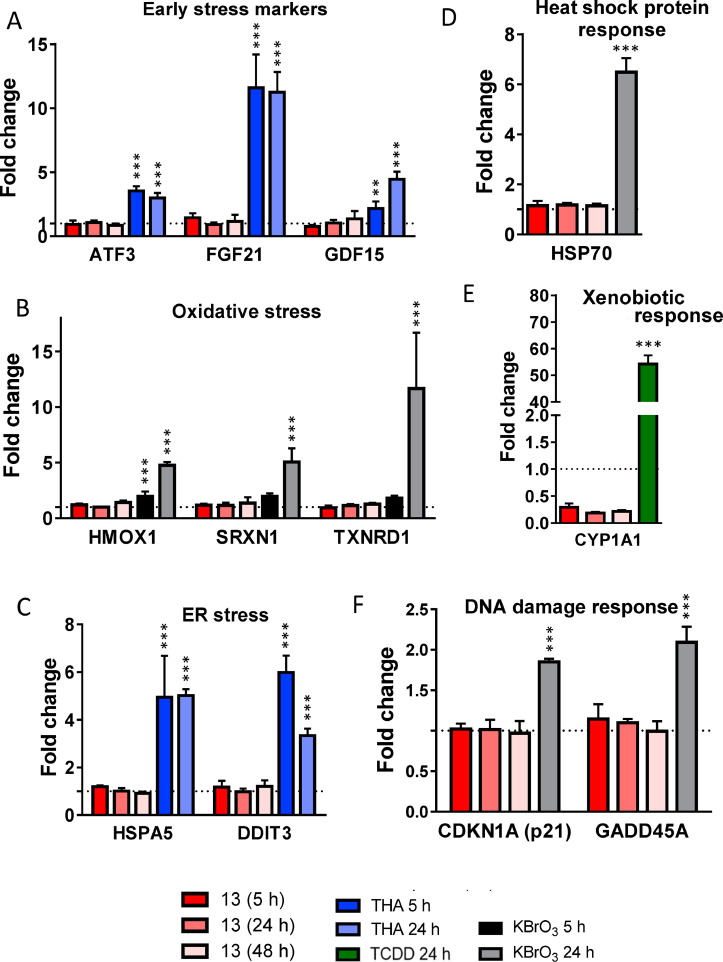
Advanced study of the cytotoxicity of compound **13**. CaCo-2 cells were seeded in 96-well plates for 24 h, then treated with **13** at 10 µM and the appropriate positive controls, and incubated for the indicated periods. Expression of the indicated stress markers was quantified by RT-qPCR. We examined the expressions of the early stress markers ATF3, FGF21, and GDF15 (A); the oxidative stress markers HMOX1, SRXN1, and TXNRD1 (B); the endoplasmic stress response markers HSPA5 and DDIT3 (C), the heat shock response protein HSP70 (D); the xenobiotic response marker CYP1A1 (E), and the DNA damage response markers CDKN1A (p21) and GADD45A (F). The means are significantly different from those of untreated cells at **P < 0.01 and ***P < 0.001. (For interpretation of the references to color in this figure legend, the reader is referred to the web version of this article.)

The markers of early toxicity—activating transcription factor (ATF3), fibroblast growth factor 21 (FGF21), and growth differentiation factor 15 (GDF15)—are activated by a number of extracellular signals and transcription factors, and thus may serve as a hub of intracellular stress signaling (Kim and Lee, 2021; Ku and Cheng, 2020; Shimizu and Sato, 2022). Compound **13** did not induce the expression of early toxicity markers at any of the tested incubation periods, in contrast to the positive control thapsigargin, which is a strong calcium homeostasis disruptor and ER stress inductor (Fig. 10A).

Moreover, compound **13** did not increase the expression of the antioxidative enzyme-encoding genes heme oxygenase 1 (HMOX1), sulfiredoxin 1 (SRXN1), or thioredoxin reductase 1 (TXNRD1). In contrast, the oxidative stress inducer KBrO_3_ had a highly stimulating effect on the expression of these genes, which was especially visible after a 24-h incubation with the tested cell culture (Fig. 10B).

Next, we determined the expression levels of genes involved in the ER stress response (also called the unfolded protein response), heat shock protein response (activated by damaged proteins in the cytoplasm), and DNA damage response. CaCo-2 cells exposed to compound **13** exhibited no changes in the expression of heat shock protein A5 (HSPA5), DNA damage inducible transcript 3 (DDIT3) (Fig. 10C), HSPA1B (HSP70) (Fig. 10D), cyclin dependent kinase inhibitor 1A (CDKN1A; p21), or growth arrest and DNA damage inducible alpha (GADD45A) (Fig. 10F). In contrast, these gene expressions were increased by the positive controls thapsigargin and KBrO_3_.

Finally, we measured mRNA level of cytochrome P450 1A1 (CYP1A1), the target gene of the xeno-sensing arylhydrocarbon receptor (AhR). Compound **13** significantly decreased CYP1A1 expression for all tested incubation periods. TCDD, a model AhR agonist, increased the CYP1A1 mRNA level up to 50-fold (Fig. 10E).

## 4 Discussion

Perylene derivatives are synthetic amphipathic structures with broad-spectrum antiviral activity against multiple enveloped and phylogenetically unrelated viral pathogens, including influenza A virus (*Orthomyxoviridae*), human parainfluenza virus type 3 (*Paramyxoviridae*), vesicular stomatitis virus (VSV, *Rhabdoviridae*), hepatitis C virus (HCV, *Flaviviridae*) (Colpitts et al., 2013), human respiratory syncytial virus (RSV, *Paramyxoviridae*) (Nikolayeva et al., 2020), tick-borne encephalitis virus (TBEV, *Flaviviridae*) (Orlov et al., 2016), African swine fever virus (*Asfarviridae*) (Hakobyan et al., 2018), and herpes simplex viruses (HSV-1, HSV-2, *Herpesviridae*) (Colpitts et al., 2013; Speerstra et al., 2018). Recently, they have also been demonstrated to be active against yellow fewer virus and SARS-CoV-2 (Chistov et al., 2023). In the present study, we describe in detail the molecular basis of the anti-SARS-CoV-2 activity of amphipathic perylene derivatives. The studied perylene compounds exhibited antiviral activity against two representatives of the *Coronaviridae* family, SARS-CoV-2 and FIPV, in various cell cultures of human or animal origin that are susceptible to coronaviral infection. Moreover, we demonstrated that perylene compounds inhibited the replication of multiple SARS-CoV-2 subvariants that have been circulating in the human population.

Due to their amphipathic character, perylene compounds show high affinity for cellular, viral, and liposomal membranes. The strong affinity of perylene compounds for membranes, together with photodynamic peroxidation of membrane phospholipids, is the predominant molecular basis for their high antiviral potency, manifested as efficient inhibition of virus–cell fusion machinery. Well-balanced amphipathicity of perylene compounds is important for efficient compound–membrane interaction, and results in strong antiviral activity. Indeed, we observed that perylene compounds with balanced molecular amphipathicity exhibited the highest anti-SARS-CoV-2 activity, whereas derivatives with either highly polar (compounds **5** and **20**) or highly hydrophobic (compounds **8** and **14**) substituents showed low/negligible anti-SARS-CoV-2 effects.

Antiviral activity was significantly lower when infection was carried out at a low temperature (4 °C). We suggest that at 37 °C, the viral envelope is fully fluid and completely amenable to the incorporation of amphipathic perylene molecules, resulting in strong inactivation of the virus. On the other hand, at 4 °C, the membranes of most enveloped viruses are rigid, which prevents the insertion of amphipathic agents into the viral envelopes (St.Vincent et al., 2010; St. Vincent et al., 2010). Furthermore, we can assume that the observed inhibitory effect was not related to the binding of perylene compounds to viral surface proteins (e.g., spike protein S1), since such interactions should be not be affected by temperature.

Using a cell-based fusion assay we demonstrated that perylene compounds directly affect the virus-cell fusion machinery and block the entry of SARS-CoV-2 into host cells. It is very interesting that perylene antivirals abort the virus–cell fusion process of multiple enveloped viruses that exhibit significantly different virus–cell entry mechanisms (Colpitts et al., 2013). For example, HSV-1 enters host cells predominantly via plasma membrane interactions and fusion at the cell surface (White and Whittaker, 2016). On the other hand, the virus–cell fusion machineries of coronaviruses and flaviviruses are strictly based on receptor-mediated endocytosis, followed by lower pH-induced fusion of the viral envelope with the endosomal membrane (Hu et al., 2017; Štefánik et al., 2022). Obviously, the perylene-based antiviral effect is independent of these differences in virus–cell entry mechanisms.

The main mechanism underlying the antiviral action of perylene compounds is based on singlet oxygen photogeneration, and the disruption of viral membrane rheology through peroxidation of membrane phospholipids. Our present results are in agreement with the findings of Vigant et al. (2014), and indicate that most perylene derivatives are extremely strong photosensitizers that can generate singlet oxygen under blue light irradiation. In this context, they are very similar to the compound LJ001 and its derivative LJ002, which were recently reported to have photosensitizing activity (Wolf et al., 2010; Zeng et al., 2020). We observed that the thiophene-containing compounds had the highest photosensitization capacity, which was in good agreement with their strong (nano-molar) anti-SARS-CoV-2 activity (particularly for compounds **17** and **18**) and high membrane penetration capacity. On the other hand, compounds **14** and **20** showed poor singlet oxygen production capacity and low affinity for membranes, which resulted in negligible antiviral activity. Interestingly, some compounds with high membrane affinity and high photosensitization capacity (e.g., **8** and **12**) exhibited no/weak anti-SARS-CoV-2 activity. This phenomenon might be related to the depth of membrane penetration, as the relatively long and hydrophobic buthylamide and hydroxyhexylamide chains of **8** and **12** likely penetrate much deeper into the lipid membranes compared to the other tested compounds. This suggests a potential influence of the distance between the membrane-integrated photosensitizer and the C=C double bond in the viral membrane phosopholipids, particularly considering the short lifetime and high reactivity of singlet oxygen.

Regarding the cytotoxicity of perylene compounds, most of the derivatives studied were well tolerated by various cell lines, including Vero, CaCo-2, and CRFK, when the experiment was performed under normal light conditions or the compounds were incubated with the cells in the dark. Similar to our results, Chistov et al. (2019) reported that numerous compounds based on the 5-(perylene-3-ylethynyl)uracil scaffold exhibited low cytotoxicity towards Vero, PEK, and RD cells. Other perylene-related compounds described by Slesarchuk et al. (2020) and St.Vincent et al. (2010) have also exhibited very low cytotoxicity when incubated with Vero and PEK cells, respectively. Although photosensitizing perylene antivirals non-selectively interact with both viral and cellular membranes, it is likely that metabolically active cells (in contrast to metabolically inert viruses) can overcome the deleterious membrane-disrupting effect through metabolic (energy-consuming) rearrangement of membrane lipids, thus restoring the physiological rheology of cellular membranes. Therefore, the interactions of most perylene compounds with cellular membranes do not result in significant cytotoxic effects, apoptotic/necrotic reactions, or decreased proliferation capacity in most cell types under normal lighting conditions (Colpitts et al., 2013).

Our study also showed that perylene-ethynyl scaffolds substituted with small charged groups, non-polar groups, or a long hydroxylated chain resulted in a moderate decrease in cell viability. In agreement with our results, Speerstra et al. (2018) reported that perylene compounds with a charged or less polar group attached to the perylene-ethynyl core exhibited increased cytotoxic/cytostatic activity in Vero cells. This phenomenon should be investigated in greater detail in future research. In contrast, the same authors demonstrated that the type of hydrophobic group (e.g., replacement of the perylene residue with a pyrene or phenyl group) did not significantly affect the toxicity of perylene derivatives (Speerstra et al., 2018). Notably, treatment with perylene compound **13** did not lead to a change in the expression of cellular stress-related genes, with the exception that CYP1A1—the target gene of the xeno-sensing arylhydrocarbon receptor (AhR)—was significantly decreased for all tested cultivation periods. We assume that the perylene ring of compound **13** interacts with AhR and changes the expression of AhR-target genes. While benzoperylenes have been previously shown to be weak partial AhR agonists (Machala et al., 2001), compound **13** exhibited a suppressive effect on AhR-dependent gene expression. CYP1A enzymes are involved in the biotransformation of xenobiotics (including aromatic environmental contaminants and methylxanthine drugs) as well as endogenous compounds (including steroids). Thus, the perylene compound-induced suppression of CYP1 enzyme expression might reduce the catabolism of steroid hormones. Since the perylenyl group is present as a functional component of perylene-based pharmacophores, these compounds must be subjected to rigorous *in vitro* mutability and carcinogenicity assays, and their ability to induce malignant transformation must be verified in an animal model. These issues will be the subject of our future studies.

However, the cytotoxicity of the perylene compounds increased dramatically after irradiation of compound-treated cells with blue light. The photosensitizer-mediated formation of ROS under blue light irradiation is significantly higher than under normal light conditions or in the dark; the increased amounts of singlet oxygen can damage cell membranes and other target structures in cells, leading to a rapid decrease in cell viability. This is not surprising, as photosensitizers have already been used as effective anticancer agents (Xiao et al., 2018).

## 5 Conclusion

Our present results clearly showed that the observed anti-SARS-CoV-2 effect of amphipathic perylene derivatives was exclusively due to compound-mediated photodynamic inactivation of SARS-CoV-2 virions (photosensitization mechanism). The biophysical (incorporation) mechanism of antiviral action of perylene compounds (based on the molecular geometry and shape of the molecule) did not play a significant role in the inhibition of SARS-CoV-2 replication induced by perylene derivatives.

This presently identified predominant mechanism of action of amphipathic perylene derivatives will considerably limit their potential application as anti-SARS-CoV-2 agents. SARS-CoV-2 was used as a model enveloped viral pathogen of high medical importance. However, the structure–activity relationships and the methodological concepts proposed in this work can be the basis for the future application of photosensitizing pharmacophores to fight pathogens that cause, for example, superficial skin or mucosal infections, such as herpesviruses. Moreover, there is a need for in-depth investigation of the potential of photosensitizing antivirals for preventing infection within the oral cavity and the respiratory tract, because many respiratory viruses are enveloped.

Overall, amphipathic perylene compounds are extremely interesting structures, with biophysical and photochemical properties that suggest their potential future use not only as ROS-generating photosensitizers, but also as membrane-specific fluorescent probes or inactivants for the preparation of non-infectious viral particles and/or light-inactivated virus-based vaccines. As such, they could be useful for applications in numerous biological and bio-medical disciplines.

## CRediT authorship contribution statement

**Petra Straková:** Methodology, Validation, Investigation, Data curation, Writing – original draft. **Petr Bednář:** Methodology, Investigation, Writing – original draft. **Jan Kotouček:** Methodology, Software, Formal analysis, Investigation, Writing – original draft. **Jiří Holoubek:** Methodology, Investigation. **Andrea Fořtová:** Formal analysis, Methodology, Investigation. **Pavel Svoboda:** Formal analysis, Investigation, Software. **Michal Štefánik:** Methodology, Investigation. **Ivana Huvarová:** Methodology, Investigation. **Pavlína Šimečková:** Conceptualization, Validation, Investigation, Writing – original draft, Supervision. **Josef Mašek:** Conceptualization, Validation, Investigation, Writing – original draft, Supervision. **Daniil A. Gvozdev:** Methodology, Validation, Formal analysis, Investigation, Writing – original draft. **Igor E. Mikhnovets:** Methodology, Validation, Formal analysis, Writing – original draft. **Alexey A. Chistov:** Methodology, Validation, Formal analysis. **Timofei D. Nikitin:** Methodology, Validation, Formal analysis, Writing – original draft. **Maxim S. Krasilnikov:** Methodology, Formal analysis, Investigation. **Alexey V. Ustinov:** Data curation, Writing – review & editing, Visualization, Supervision. **Vera A. Alferova:** Data curation, Writing – review & editing, Visualization, Supervision. **Vladimir A. Korshun:** Conceptualization, Writing – review & editing, Resources, Visualization, Supervision, Project administration, Funding acquisition. **Daniel Růžek:** Writing – review & editing, Project administration, Funding acquisition. **Luděk Eyer:** Conceptualization, Methodology, Validation, Formal analysis, Investigation, Data curation, Writing – original draft, Writing – review & editing, Visualization, Supervision, Project administration, Funding acquisition.

## Declaration of Competing Interest

The authors declare that they have no known competing financial interests or personal relationships that could have appeared to influence the work reported in this paper.

## Data Availability

Data will be made available on request.

## Appendix Supplementary materials

Image, application 1
