# Supplementary material for: Antiviral activity of singlet oxygen-photogenerating perylene compounds against SARS-CoV-2: Interaction with the viral envelope and photodynamic virion inactivation
Source: Virus Res. 2023 Jun 29;334:199158. doi: 10.1016/j.virusres.2023.199158 (PMC10307035; doi:10.1016/j.virusres.2023.199158)
Supplement: Supplementary file 1 [file mmc1.pdf]

Antiviral Activity of Singlet Oxygen-Photogenerating Perylene Compounds Against SARS-CoV-2: Interaction with the Viral Envelope and Photodynamic Virion Inactivation

Petra Straková[a,b,c], Petr Bednář[a,b,c], Jan Kotouček[d], Jiří Holoubek[a,b,c], Andrea Fořtová[a], Pavel Svoboda[a,b,c,e], Michal Štefánik[f], Ivana Huvarová[a], Pavlína Šimečková[d], Josef Mašek[d], Daniil A. Gvozdev,[g] Igor E. Mikhnovets,[h,i] Alexey A. Chistov,[i] Timofei D. Nikitin,[h,i] Maxim S. Krasilnikov,[h,i] Alexey V. Ustinov,[i] Vera A. Alferova,[i] Vladimir A. Korshun,[i] Daniel Růžek[a,b,c,], and Luděk Eyer[a,b,c,*]

[a]Veterinary Research Institute, Emerging Viral Diseases, Hudcova 296/70, CZ-621 00 Brno, Czech Republic

[b]Institute of Parasitology, Biology Centre of the Czech Academy of Sciences, Branišovská 1160/31, CZ-370 05 České Budějovice, Czech Republic

[c]Department of Experimental Biology, Faculty of Science, Masaryk University, CZ-62500 Brno, Czech Republic

[d]Veterinary Research Institute, Pharmacology and Toxicology, Hudcova 296/70, CZ-621 00 Brno, Czech Republic

[e]Department of Pharmacology and Pharmacy, Faculty of Veterinary Medicine, University of Veterinary Sciences Brno, Palackého tř. 1946/1, 612 42 Brno-Královo Pole, Czech Republic

[f]Department of Chemistry and Biochemistry, Mendel University in Brno, CZ-61300 Brno, Czech Republic

[g]Department of Biology, Lomonosov Moscow State University, Moscow, 119991, Russia

[h]Department of Chemistry, Lomonosov Moscow State University, Moscow, 119991, Russia

[i]Shemyakin-Ovchinnikov Institute of Bioorganic Chemistry, Moscow, 117997, Russia

*Corresponding author at: Veterinary Research Institute, Emerging Viral Diseases, Hudcova 296/70, CZ-621 00 Brno, Czech Republic, e-mail address: ludek.eyer@vri.cz (L. Eyer).

1. Supplementary material and methods

1.1. Fluorescence quantum yield (QY) determination

The steady-state fluorescence and absorption data were used for quantum yield determination. QY was determined relative to a reference compound, rhodamin B. Absorbance in the range of 0.01–0.1 was used to calculate the QY. We plotted two calibration curves of integrated emission fluorescence intensity as a function of sample absorbance. QY was obtained using the corresponding gradient, according to equation 1 (Kozma et al., 2005; Magde et al., 1999; Williams et al., 1983).

$$QY_s = QY_r \cdot \left(\frac{A_s}{A_r}\right) \cdot \left(\frac{n_s}{n_r}\right)^2 \tag{1}$$

In equation 1, QY represents quantum yield, A the gradient, and n the refractive index of the solvent, the subscript "*s*" represents *sample*, and the subscript "r" represents *reference*. The standard value of QY_r for rhodamin B is 31% (Water; λ = 514 nm) (Magde et al., 1999). The refractive index of DMSO is 1.4772, and the refractive index for Milli-Q water is 1.33 (Kozma et al., 2005).

1.2. Preparation of liposomes

For the kinetic study of the incorporation of selected compounds into membranes, a suspension of unilamellar liposomes was prepared using the film hydration method, according to the following protocol. The required amount of lipids for the liposomal composition (EPC/cholesterol 70/30 mol%) was dissolved in chloroform at a total lipid concentration of 1 mg/mL. The suspension was transferred into a round-bottom flask. With a constant temperature (corresponding to the transitive temperature of the used lipid), reduced pressure, and rotation, the organic solvent was evaporated to form a thin lipid film. Next, this lipid film was hydrated

using PBS (pH 7.4), and the resulting suspension was repeatedly frozen and thawed in liquid N_2 (5 cycles), and subsequently extruded using a manual Avanti® Mini-Extruder and polycarbonate membrane filters (Whatman Nuclepore) with 200-nm pore size.

The quality of the liposomal suspension was evaluated using the Multi-Angled Dynamic Light Scattering (MADLS®) technique. The required amount of the suspension was placed in a low-volume ZEN2112 quartz batch cuvette (Malvern Panalytical Ltd, Malvern, UK) and measured using a Zetasizer Ultra (Malvern Panalytical Ltd, UK) at a constant temperature of 25°C. The device was equipped with a HeNe Laser (633 nm) and three detectors at the following angles: 173° (backscatter), 90° (side scatter), and 13° (forward scatter). The measured data were evaluated using ZS Xplorer software (Malvern Panalytical Ltd, UK). The measured values of hydrodynamic size, polydispersity index (PdI), and concentration are reported as mean value ± standard deviation (n = 3).

Incorporation kinetics were determined using steady-state fluorescence spectroscopy at a constant excitation and emission wavelength, according to the corresponding sample excitation and emission maxima. Samples were diluted with PBS to a final concentration of 0.1 mM. After homogenization, the corresponding fluorescence intensity was measured for 150 s. Then, 50 µL of LNP suspension was added to the mixture, and the increase of fluorescence intensity was monitored during the period of 150–1400 s in the same setup (Fig. S2).

1.3. Design of the blue light irradiation system

To study the light-induced antiviral activity of perylene compounds (photodynamic virion inactivation), a special irradiation system was developed and adapted to the 96-well plate format. The device comprises 96 LED diodes wired in 8 parallel branches, with each LED illuminating one well. The utilized LED diodes have a dominant wavelength of 465–480 nm, luminous intensity of 200–390 mcd (If = 20 mA), and a viewing angle of 60–70°. The device

was connected to a power supply of constant DC current (PowerPac™ HC High-Current Power Supply/Bio-Rad) and powered by constant 30 mA (30 mW/cm^2 power density). It is assumed that all emitted photons go through the bottom of the well, accounting for the viewing angle of the LED, and the distance from the well bottom to the LED.

2. Supplementary references

Kozma, I.Z., Krok, P., Riedle, E., 2005. Direct measurement of the group-velocity mismatch and derivation of the refractive-index dispersion for a variety of solvents in the ultraviolet. Journal of the Optical Society of America B 22. https://doi.org/10.1364/JOSAB.22.001479

Magde, D., Rojas, G.E., Seybold, P.G., 1999. Solvent Dependence of the Fluorescence Lifetimes of Xanthene Dyes. Photochem Photobiol 70. https://doi.org/10.1111/j.1751-1097.1999.tb08277.x

Williams, A.T.R., Winfield, S.A., Miller, J.N., 1983. Relative fluorescence quantum yields using a computer-controlled luminescence spectrometer. Analyst 108. https://doi.org/10.1039/an9830801067

3. Supplementary tables

Supplementary Table 1. Solubility of perylene compounds in 15% DMSO.

Compound No.	Compound Abbreviation	**Solubility in 15% DMSO [M]**
**1**	aUY11	$1.5 \cdot 10^{-5}$
**2**	dUY11	$7.8 \cdot 10^{-6}$
**3**	UY11	$3.6 \cdot 10^{-5}$
**4**	m_2UY11	$1.3 \cdot 10^{-4}$
**5**	cm1pUY11	$2.9 \cdot 10^{-3}$
**6**	cm2pUY11	$5.3 \cdot 10^{-6}$
**7**	cm3pUY11	$6.4 \cdot 10^{-4}$
**8**	cm4pUY11	$3.0 \cdot 10^{-6}$
**9**	cm6pUY11	$4.5 \cdot 10^{-4}$
**10**	cm7pUY11	$4.9 \cdot 10^{-5}$
**11**	cm8pUY11	$9.0 \cdot 10^{-4}$
**12**	cm9pUY11	$9.4 \cdot 10^{-4}$
**13**	cm22pUY11	$3.0 \cdot 10^{-3}$
**14**	Me_2NPY11	$2.4 \cdot 10^{-5}$
**15**	AcNPY11	$2.5 \cdot 10^{-6}$
**16**	C1T11	$5.7 \cdot 10^{-4}$
**17**	C4T11	$2.9 \cdot 10^{-4}$
**18**	C11T11	$3.6 \cdot 10^{-6}$
**19**	C13T11	$5.9 \cdot 10^{-5}$
**20**	C11	n.d.

n.d. – not determined

4. Supplementary figures

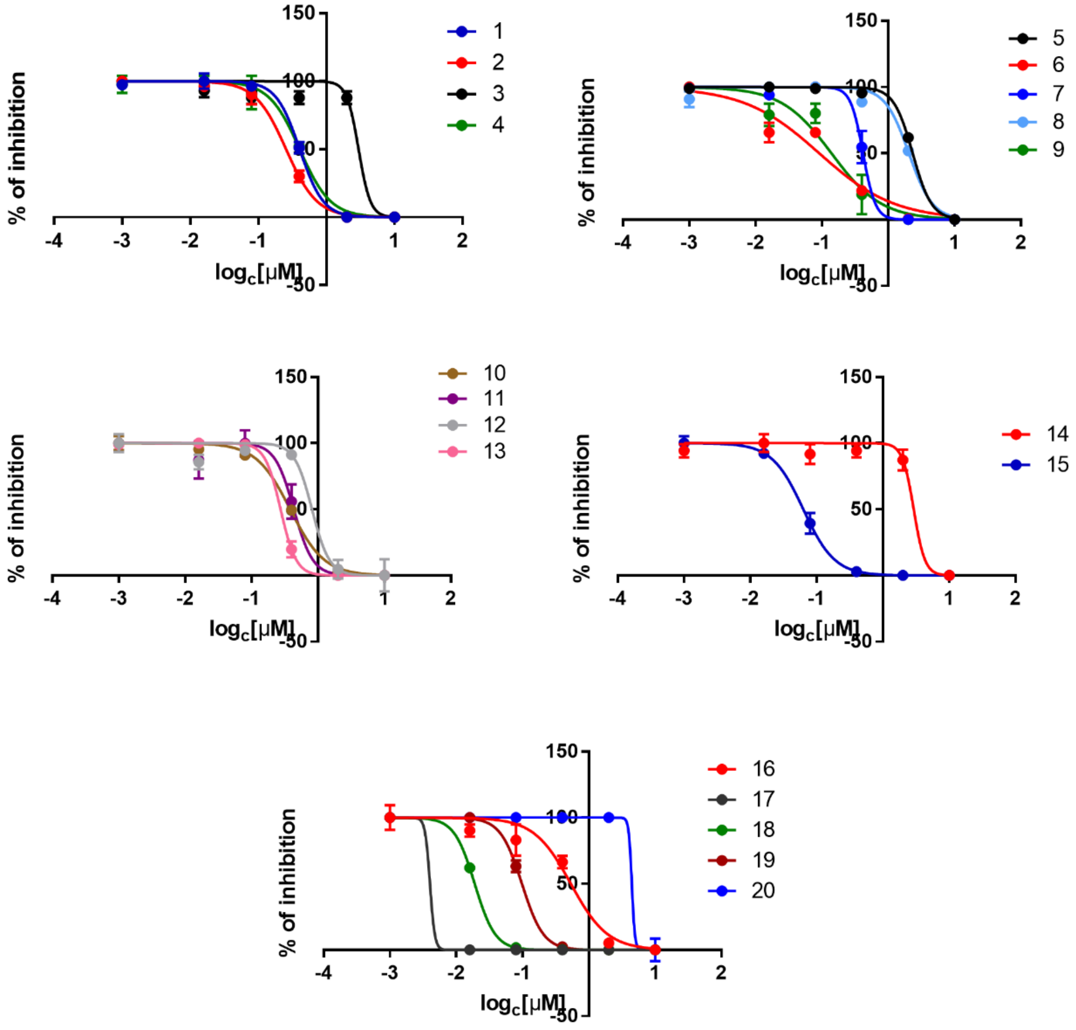

Fig. S1. Dose-response inhibition curves obtained from raw virus titers normalized and recalculated to the percentage of inhibition values. These curves were used for the estimation of EC_{50} values (Table 1). On the other hand, the App. EC_{50} values can be assessed directly from Fig. 2C–F.

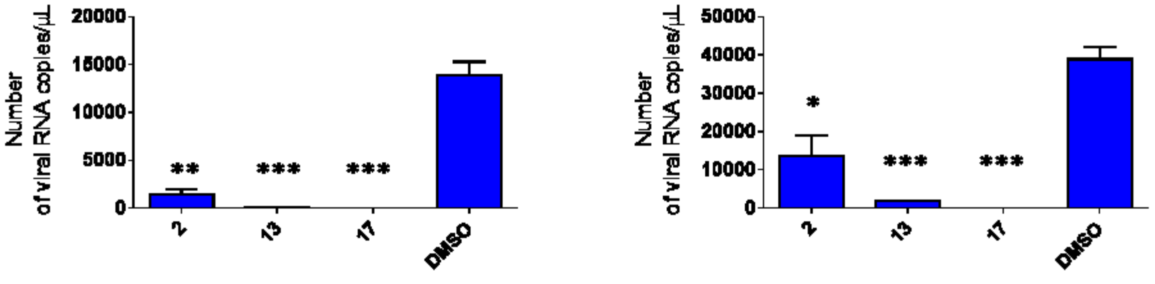

Fig. S2. Viral genomic RNA was quantified by RT-qPCR. Viral RNA was isolated from the supernatant medium (free viral RNA, left panel) or from the infected cells (intracellular viral RNA, right panel). Data are expressed as the mean ± SEM of two independent experiments, each performed in triplicate. The means are significantly different from those of virus-infected DMSO-treated cells at **P* < 0.05, ***P* < 0.01, and ****P* < 0.001.

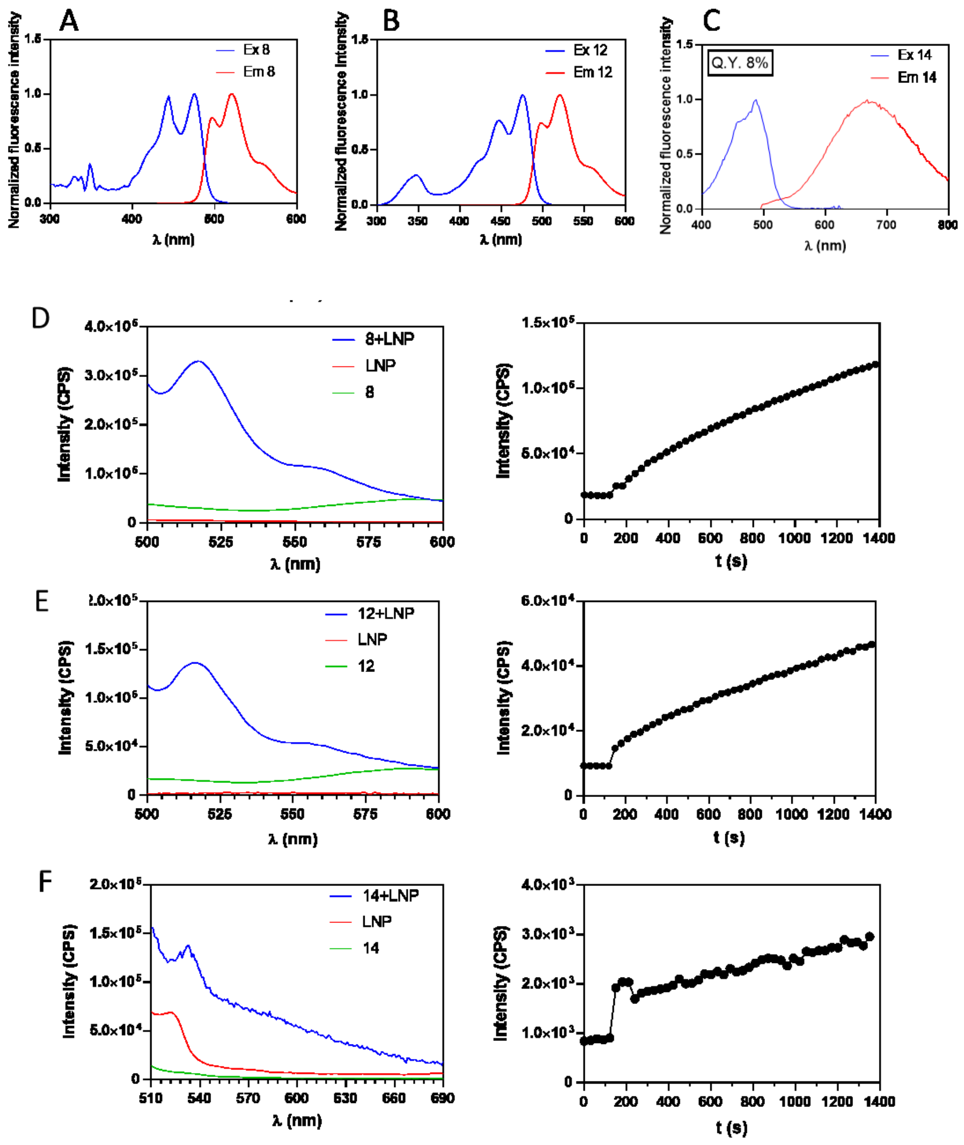

Fig. S3. Fluorescence spectra and penetration of selected compounds **8**, **12**, and **14** into liposomes. (A–C) Ex/Em spectra of the compounds in DMSO, and the determined fluorescence quantum yields. (D–F) Fluorescence spectra and the kinetics of penetration of compound **8** (D), compound **12** (E), and compound **14** (F). Left panels: Fluorescence spectra of free compounds in PBS (10 µM, green lines), free liposomes (LNP, red lines), and a mixture of a compound and LNP in PBS (blue lines). Right panels: Kinetics of penetration of the compounds (10 µM) into liposomes, measured at 520 nm for compounds **12** and **8**, and at 540 nm for compound **14**.

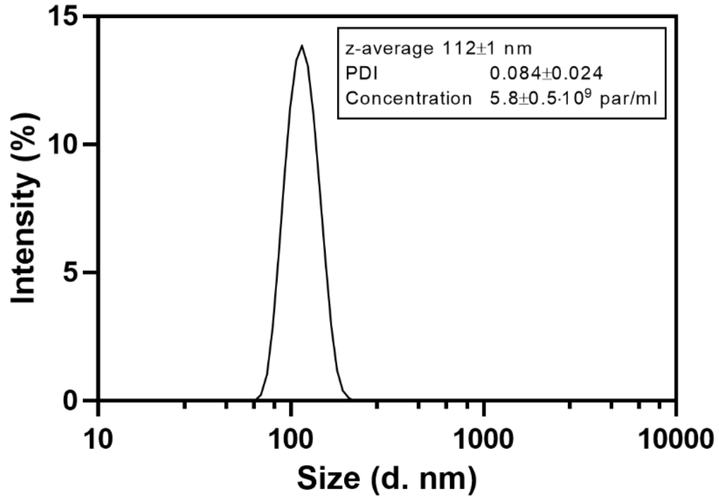

Fig. S4. Evaluation of the size (diameter in nm) of the prepared liposomes (LNP).

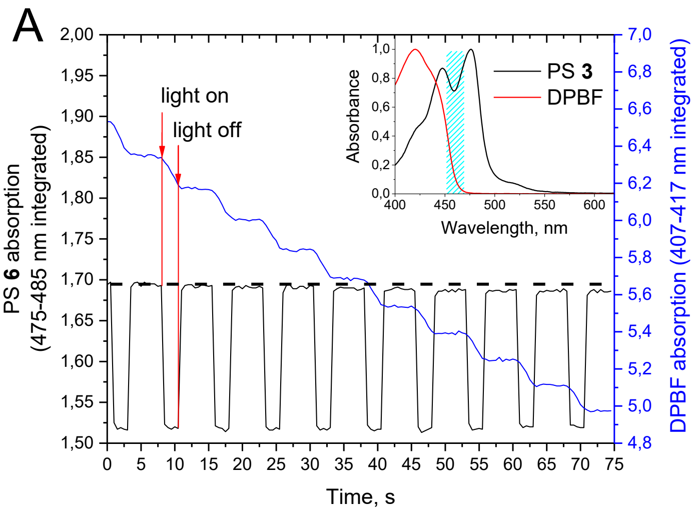

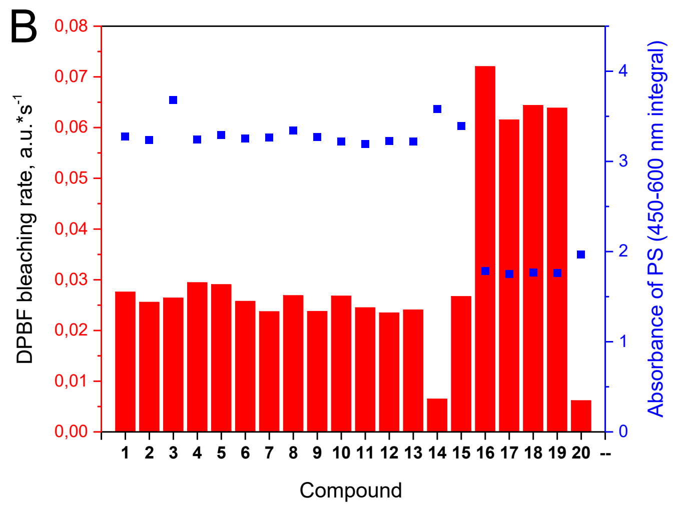

Fig. S5. Quantification of the reactive oxygen species (ROS) generation rate. (A) Changes in the optical density in the absorption maximum of DPBF and the long-wavelength absorption band of compound **6** upon impulse irradiation (1 s "on" – 5 s "off") of a PS-DPBF solution in the 450–470 nm range. Dotted curve shows the absorbance of compound **6** before illumination. Inset shows the normalized absorption spectra of DPBF and compound **3**. Cyan shading indicates the area of the sample irradiation. (B) The bleaching rate (red) of DPBF in methanol with 5 μM of individual perylene compounds under light irradiation in the region of 450–600 nm, and area (blue) under the absorption spectrum of the PS (1 μM) in the 450–600 nm range.

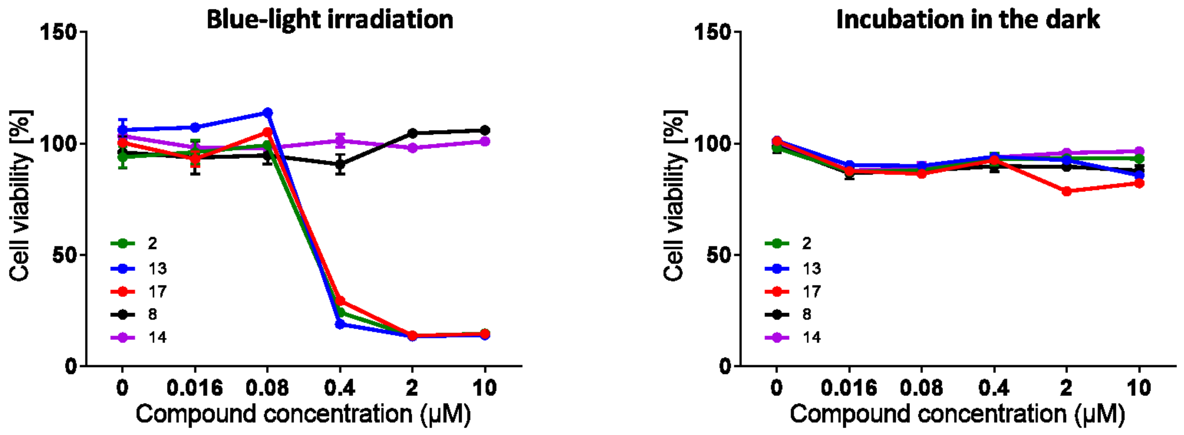

Fig. S6. Cytotoxicity of perylene compounds after irradiation of compound-treated cells with blue light. Vero cells were cultured for 24 h in 96-well plates and treated with the tested compounds at the indicated concentrations. Then, the compound-treated cells were irradiated for 10 min at RT with LEDs (465–480 nm, 30 mW/cm^2) (left panel). As a negative control, compound-treated cells were incubated for 10 min in the dark at RT (right panel). Subsequently, both irradiated and non-irradiated cell monolayers were incubated in the dark at 37°C for 24 h. Cytotoxicity was determined based on cell viability using Cell Counting Kit-8 (Dojindo Molecular Technologies, Munich, Germany). Data are expressed as the mean ± SD of two independent experiments, each performed in triplicate.

A

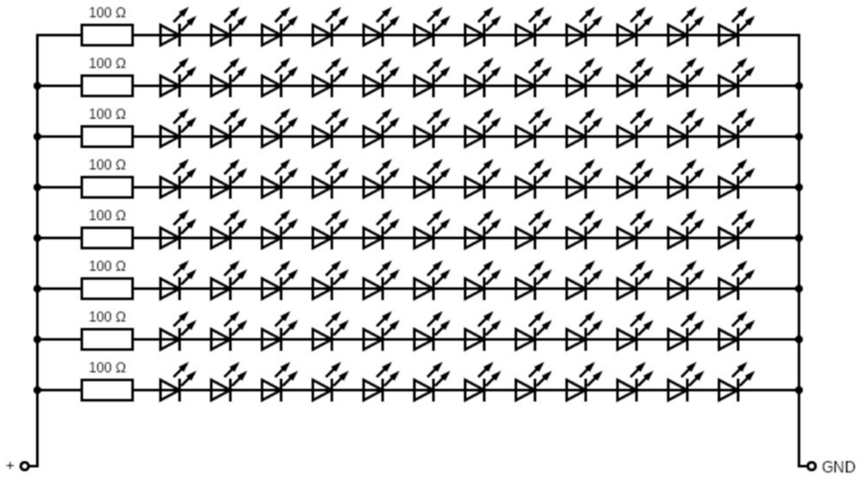

B

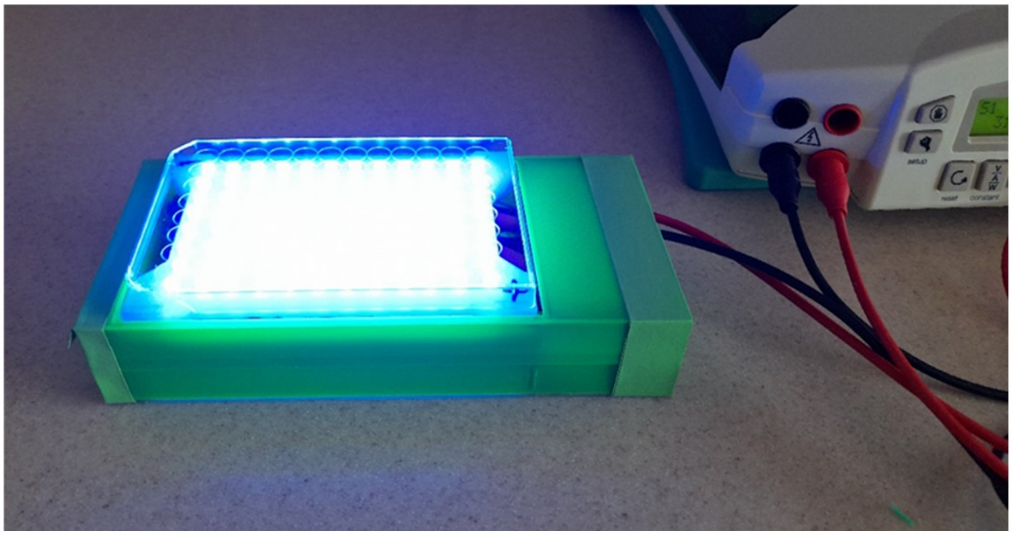

Fig. S7. Design of the blue light irradiation system. (A) Schematic representation (wiring diagram) (A) and a photograph (B) of the irradiation system used in this study.
